# Programmable engineered bacteria manipulate metabolism and remodel the TME *in situ* for enhancing adoptive cell therapy

**DOI:** 10.1016/j.ymthe.2026.06.042

**Published:** 2026-07-09

**Authors:** Jin Chen, Tianliang Liu, Xiumin Liu, Hongyue Zhang, Chen Wang, Yuan Meng, Miaoqing Wu, Sachiyo Nomura, Zhe Zhang, Songcheng Yin, Changhua Zhang, Aoran Dong

**Affiliations:** 1 Department of Medical Oncology, Fudan University Shanghai Cancer Center, Shanghai 200032, China; 2 College of Pharmaceutical Sciences, Soochow Medical College of Soochow University, Soochow University, Suzhou 215123, China; 3 Shanghai Key Laboratory of Cancer Systems Regulation and Clinical Translation, Jiading District Central Hospital, Renji Hospital Jiading Branch, Shanghai 201822, China; 4 Digestive Diseases Center, The Seventh Affiliated Hospital Sun Yat-sen University, Shenzhen, Guangdong 518107, China; 5 LifeX Institute, School of Medical Technology, Gannan Medical University, Ganzhou, Jiangxi 341000, China; 6 Henan University of Science and Technology, Luoyang, Henan 471023, China; 7 Department of Gastrointestinal Surgery, Graduate School of Medicine, The University of Tokyo, Tokyo, Japan

**Keywords:** D-mannose, adoptive cell therapy, metabolism, tumor membrane vesicles, solid tumors

## Abstract

In solid tumors, the efficacy of adoptive T cell therapy (ACT) is limited by a metabolically constrained tumor environment that undermines T cell persistence, fitness, and infiltration. Here, we engineered a hypoxia-activated bacterial hybrid system (MM@TMV) to address these barriers. This hybrid system integrates metabolically engineered bacteria and tumor membrane vesicles (TMVs) to achieve tumor-restricted metabolic reprogramming and immune reinforcement within hypoxic tumors. Within hypoxic tumors, D-mannose is produced *in situ* to support stem-like phenotypes and limit exhaustion, while TMVs facilitate both direct and APC-mediated activation of CAR-T and TCR-T cells, concurrently restraining tumor cell growth. In orthotopic, refractory, and metastatic tumor models, MM@TMV further improved T cell persistence, enhanced intratumoral infiltration, and achieved sustained tumor suppression. In a humanized patient-derived xenograft model of Claudin18.2-positive gastric tumors, MM@TMV similarly potentiated clinically relevant ACT. Biosafety tests confirmed systemic safety. Collectively, our findings establish engineered bacteria as programmable immune metabolic modulators that enable effective and safe ACT in solid tumors.

## Graphical abstract

**Figure:**
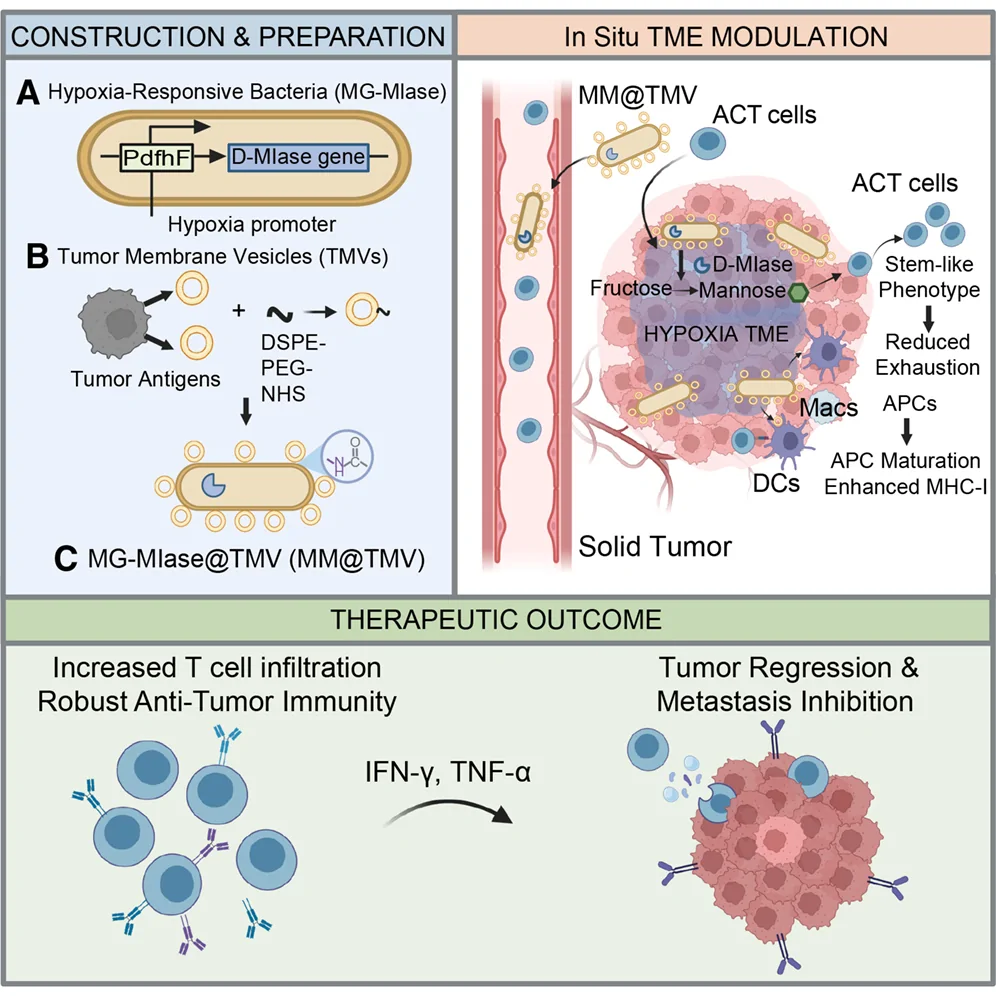

## Teaser

A hypoxia-responsive bacteria-vesicle hybrid platform generates mannose within tumors while enhancing antigen presentation, thereby preserving T cell stemness and improving adoptive cell therapy in solid tumors. This study highlights a strategy that couples metabolic rewiring with immune reinforcement to overcome key microenvironmental barriers to cellular immunotherapy.

## Introduction

Adoptive T cell therapy (ACT), such as chimeric antigen receptor (CAR) T cell therapy, has achieved remarkable success in the treatment of hematological malignancies.^1^^,^^2^^,^^3^ However, its therapeutic efficacy in solid tumors remains limited, and most patients still lack effective and durable treatment options, underscoring a major unmet clinical need.^4^^,^^5^ The poor performance of ACT in solid tumors is primarily attributed to the highly immunosuppressive tumor microenvironment (TME), in which ACT cells tend to enter a state of exhaustion.^6^^,^^7^ This exhausted state leads to ACT cell dysfunction and poor *in vivo* persistence.^8^^,^^9^ Moreover, the ability of antigen-presenting cells (APCs) to uptake, process, and present antigens is impaired in the TME. Additionally, insufficient intratumoral infiltration of ACT cells prevents them from exerting effective antitumor activity.^10^ Despite ongoing efforts to optimize CAR-T cell design, the majority of next-generation engineering strategies remain at a preclinical or early translational stage and have yet to overcome the fundamental metabolic and immunological barriers imposed by the tumor environment.^11^^,^^12^ Collectively, these factors represent key challenges that limit the effectiveness of ACT cell immunotherapy.^13^^,^^14^

Currently, metabolic modulation has emerged as a pivotal strategy for reshaping the tumor immune microenvironment and enhancing the efficacy of ACT.^15^^,^^16^ The metabolic state of the TME plays a crucial role in determining immune cell function, particularly T cell exhaustion, function, and persistence, which are directly influenced by metabolic regulation. Recent studies have shown that D-mannose, a simple hexose, can modulate immune cell function through various mechanisms, exerting significant antitumor effects. By regulating immune cell metabolism, D-mannose helps to maintain T cell stemness and delays their transition to an exhausted state.^17^^,^^18^^,^^19^^,^^20^^,^^21^^,^^22^^,^^23^^,^^24^ However, despite the ability of orally administered D-mannose to reach tumors through the bloodstream, its accumulation and functional impact within the tumor remain limited. Furthermore, D-fructose consumption is high in modern diets, but most tumors are unable to effectively metabolize D-fructose to drive glycolysis, leading to an accumulation of D-fructose within the tumor microenvironment （TME）.^25^^,^^26^ D-mannose isomerase (D-MIase), an enzyme that catalyzes the reversible conversion between D-fructose and D-mannose, provides a metabolic basis for regulating immune cell function and the TME.^27^ Therefore, we propose to utilize D-MIase to generate D-mannose directly *in situ* within the tumor.

Bacteria, such as *Escherichia coli* and *Salmonella*, have been reported to naturally possess the ability to preferentially colonize hypoxic tumor tissues, making them a promising vector for local therapeutic delivery.^28^^,^^29^ However, *E. coli* and *Salmonella* do not inherently synthesize or secrete D-MIase. Advances in genetic engineering further enable precise bacterial reprogramming, allowing controlled gene expression to manipulate the TME.^30^^,^^31^^,^^32^^,^^33^ Such engineered bacteria offer a powerful means to precondition and modulate the TME, potentially enhancing therapeutic outcomes.

In addition to metabolic modulation, the regulation of T cell function by APCs is also critical for enhancing the efficacy of ACT.^34^^,^^35^ Effective T cell activation relies not only on a supportive metabolic environment but also on antigen presentation, which modulates T cell responses. The presentation of tumor-associated antigens (TAAs) to T cells is fundamental for driving immune recognition and response, ensuring T cell activation and cytotoxic activity against tumor cells. Tumor cell membrane vesicles (TMVs) play multiple important roles in tumor immunology. TMVs retain a complete repertoire of tumor cell membrane antigens and serve as a natural source of TAAs to elicit antitumor immune responses.^36^^,^^37^ When TMVs are taken up by APCs, such as dendritic cells, TMVs can effectively promote APC maturation and activation, thereby triggering T cell activation and enhancing the ability of CD8^+^ cytotoxic T lymphocytes to recognize and eliminate tumor cells. Furthermore, TMVs could be engineered to co-deliver immune adjuvants or be combined with immunotherapeutic strategies (such as CAR-T cells or immune checkpoint inhibitors), thereby improving the TME and increasing immune cell infiltration and function within tumors.

In this study, we report a metabolically engineered vesicle-bacteria hybrid system designed to enhance the efficacy of ACT against solid tumors. This system is based on a genetically engineered, hypoxia-responsive bacterial strain modified with TMVs. Upon intratumoral (*i.t.*) or intravenous (*i.v.*) administration, the hybrid system selectively colonizes tumor tissues and initiates the synthesis of D-MIase under hypoxic TME, catalyzing the conversion of excess D-fructose into D-mannose. This metabolic modulation reprograms tumor metabolism and modulates T cell phenotypes. TMVs, carrying a full repertoire of tumor antigens, activate intratumoral ACT cells and enhance antigen presentation, thereby reshaping the TME. This hybrid system effectively addresses the challenges of limited persistence and poor infiltration of TCR-T and CAR-T cells in solid tumors and demonstrates promising therapeutic efficacy across various tumor types, highlighting its translational potential for clinical applications in solid tumor immunotherapy.

## Results

### Design and construction of hypoxia-activated bacteria

**Figure 1:**
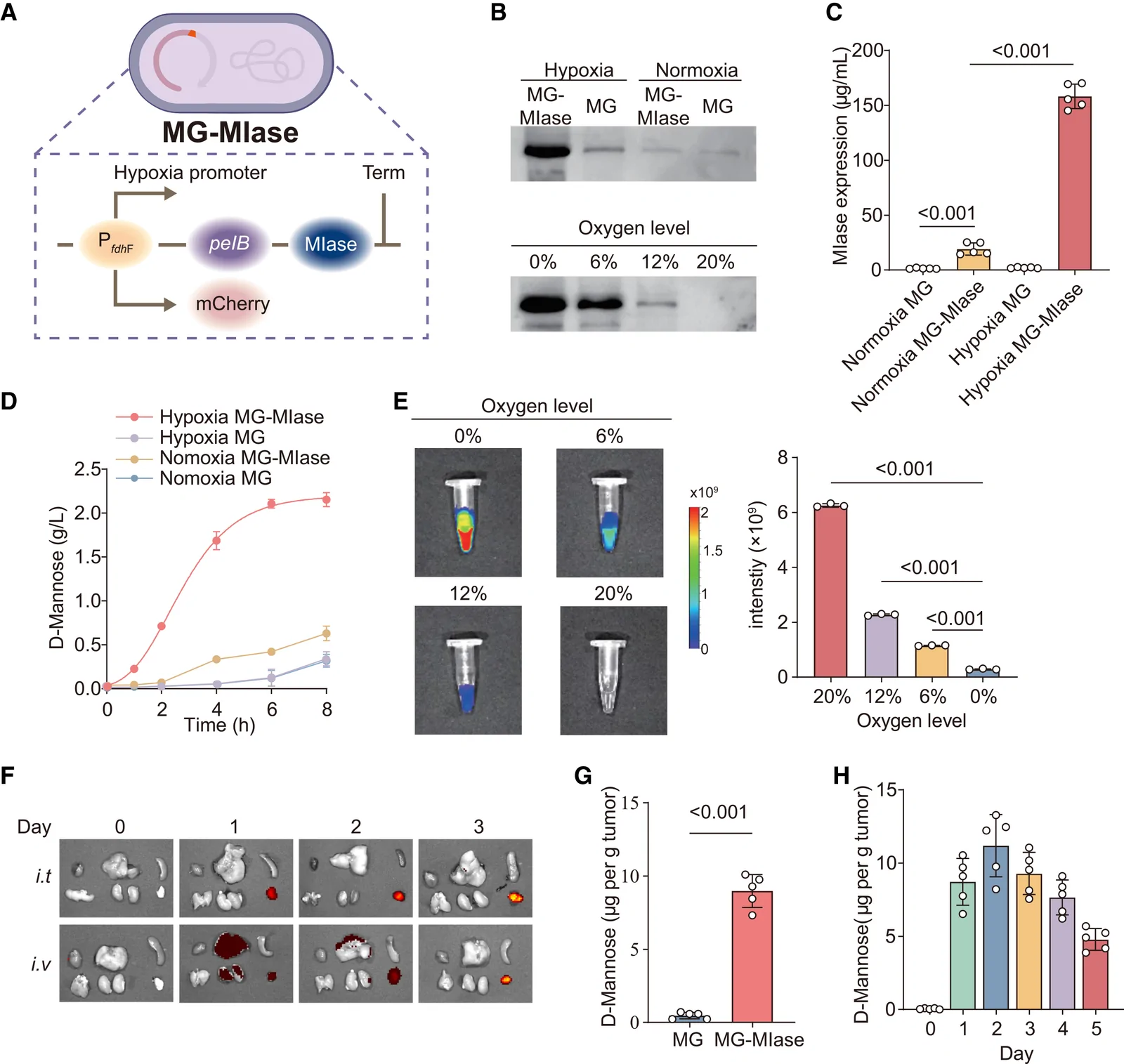
Construction and characterization of MG-MIase (A) Schematic diagram of the expression of D-MIase or mCherry in engineered bacteria in response to hypoxic environments. (B) Representative western blot images showing D-MIase protein expression in MG-MIase under different oxygen levels. The images are representative of three independent experiments. (C) D-MIase levels in the supernatant were quantified by ELISA. *n* = 5 samples/group. (D) The catalytic ability of MG-MIase to convert D-fructose into D-mannose. *n* = 5 samples/group. (E) Representative fluorescence images and quantitative analysis of *mCherry* fluorescence in MG-mCherry under different conditions. *n* = 3 samples/group. (F) Representative fluorescence images showing the colonization of MG-MIase in multiple organs following intratumoral or intravenous injection. (G and H) MG-MIase was injected into the tumor, and D-mannose levels in tumor tissue homogenates were quantified at different time points. *n* = 5 samples/group. *p* values were calculated using one-way ANOVA with Tukey’s multiple comparison test (C, E, and H) and two-way ANOVA following Fisher’s LSD test for multiple comparisons (D). Data are mean ± SEM.

To enable selective production of D-mannose within hypoxic tumors, we engineered an attenuated *Escherichia coli MG1655* strain (MG) to overexpress D-MIase, resulting in the engineered strain MG-MIase. This strain expresses D-MIase under the control of oxygen-responsive promoter *fdhF* (PfdhF). D-MIase plays a crucial role in the conversion between D-fructose and D-mannose, thereby enabling the bacteria to convert available substrate into D-mannose specifically within hypoxia tumors. An N-terminal *pelB* directs periplasmic export, while an in-frame *mCherry* fusion provided a real-time readout (Figure 1A; Figure S1A; Table S1). To validate the hypoxia responsiveness of MG-MIase, we analyzed the expression of D-MIase under different hypoxic incubation conditions. The results of western blotting (WB) showed that D-MIase expression was significantly increased under hypoxic conditions, whereas it was almost undetectable under normoxic conditions. Moreover, the increased D-MIase expression correlated with higher levels of hypoxia (Figure 1B). To further investigate the function of the engineered strain, we measured D-MIase enzymatic activity and found that it increased from 19.26 μg/mL under normoxia to 158.3 μg/mL under hypoxia (Figure 1C; *p* < 0.001). Consistently, we found that under hypoxic conditions, MG-MIase produced substantial amounts of D-mannose, indicating effective conversion of the supplied D-fructose substrate (Figure 1D). In contrast, D-mannose was undetectable in normoxic cultures or in control strains lacking the D-MIase gene. Furthermore, fluorescence intensity analysis showed that the *mCherry* signal paralleled D-MIase protein levels detected by WB, confirming that the reporter accurately reflects enzyme expression (Figure 1E). To further characterize the dynamics of hypoxia-induced enzyme production, we tracked D-MIase expression via *mCherry* fluorescence over time. Fluorescence signals emerged within 30 min of hypoxic exposure, plateaued after 2 h, and persisted for at least 5 days, indicating rapid onset and durable expression (Figures S1B and S1C).

To assess *in vivo* tumor targeting and biodistribution, we administered 1 × 10^7^ CFU of MG-MIase to 4T1-bearing mice either intravenously or intratumorally. In both delivery models, a strong fluorescence signal was already concentrated in the tumors at the earliest time point. After *i.v.* injection, a weak signal appeared in the liver and kidneys within the first day but disappeared by day 3. In contrast, the tumor fluorescence signal gradually increases over time. Meanwhile, no fluorescence signal was observed in any mouse organs after *i.t.* injection (Figure 1F). We also collected various mouse tissues at different time points, homogenized them, and plated the samples onto lysogeny broth（LB）agar to assess the number of bacterial colonies. Quantification of colony-forming units revealed that bacteria were gradually cleared from major organs such as the heart, liver, spleen, kidney, and lung. In contrast, the colony-forming units within tumors increased over time (Figures S2A and S2B). The above results suggest that the *in vivo* distribution of bacteria is hypoxia-tropic, allowing them to specifically target tumors with hypoxic properties.

We next examined the *in vivo* ability of MG-MIase to convert D-fructose into D-mannose. The D-mannose concentrations in tumors were quantified at different time points. The D-mannose content in tumors from the MG-MIase group was 15-fold higher than that in the MG group, which persisted for up to 5 days, suggesting that the engineered bacteria provide sustained D-mannose levels within the TME (Figures 1G and 1H). Overall, we successfully constructed engineered MG-MIase, which can respond to hypoxia and secrete D-MIase to synthesize D-mannose *in situ* within tumors. MG-MIase bacterial preferentially colonized tumors while being cleared from other organs following *i.v.* and *i.t.* injection.

### Engineered bacteria mediates tumor suppression through the production of D-mannose

Tumor cells depend heavily on glucose metabolism, which represents a metabolic vulnerability. Mannose competes with glucose for the same transporters, and prior studies have shown that this competition reduces glucose uptake and inhibits tumor cell growth.^20^ We therefore examined whether engineered bacteria-derived D-mannose could directly suppress tumor cells. We co-incubated four murine tumor cell lines with engineered bacteria and counted the tumor cell proliferation at different time points. MG-MIase significantly inhibited the growth of B16F10 cells, with an effect comparable to the D-mannose-supplemented control (Figure S3A). Moreover, using a panel of cell lines, we observed that the inhibitory effect of D-mannose occurred in tumor cells from various tissues with the effect being greater in some cells than others (Figure S3A). We next asked whether metabolic interference also modifies immune-evasive signaling. PD-L1 surface expression was reduced in all four tumor cell lines upon MG-MIase co-culture, with a decrease ranging from 20% to 50% relative to control (Figure S3B). Together, these findings indicated that D-mannose derived from MG-MIase limits cell-autonomous proliferation and simultaneously decreases PD-L1, potentially sensitizing tumors to immunotherapy. We then determined whether D-mannose produced by engineered bacteria could inhibit tumor growth *in vivo* without interference from the immune system. Accordingly, three irradiated and immune-ablated tumor-bearing mouse models received either a single i.t. dose (5 × 10^7^ CFU) of MG or MG-MIase, or oral D-mannose supplementation was added to the drinking water (20%). This treatment did not affect the body weight of the mice (Figures S3C–S3E). Notably, *i.t.* injection of MG-MIase delayed B16F10 outgrowth relative to the MG control, whereas neither 4T1 nor Panc-01 tumors responded appreciably (Figures S3C–S3E). These data indicated that, in the absence of adaptive immunity, metabolic competition alone affords weak suppression of tumor progression.

### MG-MIase treatment promotes stem-like T cell phenotype *in vitro*

**Figure 2:**
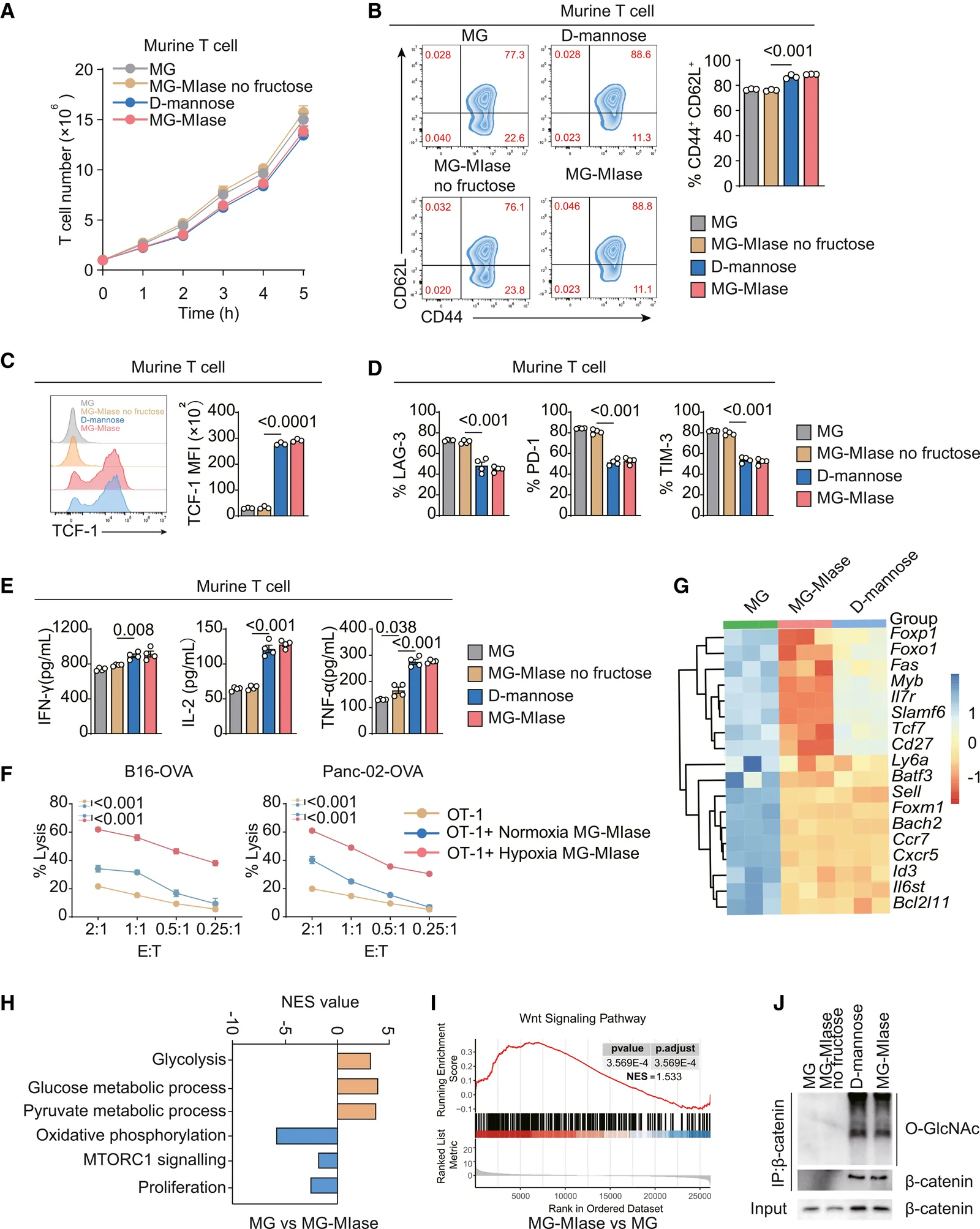
The effect of MG-MIase treatment on murine T cells *in vitro* (A) The numbers of murine T cells expanded after MG, D-mannose (20 nM), MG-MIase without D-fructose, or MG-MIase treatment. *n* = 4 samples/group. (B) Representative flow cytometry plots and quantitative analysis of CD44^+^CD62L^+^ expression in murine T cells after co-culture with MG, D-mannose, MG-MIase without D-fructose, or MG-MIase. *n* = 3 samples/group. (C) Representative flow cytometry plots and quantitative analysis of TCF1 expression in murine T cells after co-culture with MG, D-mannose, MG-MIase without D-fructose, or MG-MIase. *n* = 3 samples/group. (D) Quantification of exhaustion marker expression in murine T cells after co-culture with MG, D-mannose, MG-MIase without D-fructose, or MG-MIase. *n* = 4 samples/group. (E) Quantification of IFN-γ, TNF-α, and IL-2 expression in murine T cells after co-culture with MG, D-mannose, MG-MIase without D-fructose, or MG-MIase. *n* = 4 samples/group. (F) Under different oxygen conditions, the cytotoxicity of OT-I CD8^+^ T cells with or without MG-MIase treatment was evaluated by co-culturing them with B16-OVA or Panc-02-OVA cells at various effector (E) to target (T) ratios. *n* = 4 samples/group. (G) The heatmap showed the differentially expressed genes (DEGs) related to stemness in T cells under MG, MG-MIase, or D-mannose treatment conditions. (H) Enrichment analysis of metabolic pathways from RNA-seq data of T cells treated with MG or MG-MIase. (I) GSEA analysis of the Wnt pathway signature from RNA-seq data of T cells treated with MG or MG-MIase. (J) WB analysis of the O-GlcNAcylated level of β-catenin in OT-I T cells cultured in indicated conditions. *p* values were calculated using unpaired two-sided Student’s *t* test (B–E) and two-way ANOVA following Fisher’s LSD test for multiple comparisons (A and F). Data are mean ± SEM. *n.s.*, not significant.

Metabolic competition alone proved insufficient to restrain tumor progression. However, D-mannose has been reported to enhance T cell metabolism, promote stem-like differentiation, and reduce exhaustion.^23^ This prompted us to investigate whether D-mannose produced by MG-MIase could reprogram T cell metabolism and influence differentiation during *in vitro* expansion. In murine T cells, MG-MIase-derived D-mannose modestly inhibited expansion (6.700 × 10^6^ vs. 5.595 × 10^6^, *p* < 0.001) (Figure 2A). A similar proliferation impairment was observed in human T cells (Figure S4A). Despite the modest proliferation, MG-MIase increased the proportion of CD44^+^CD62L^+^ central memory T cells (T_CM_) by 1.2-fold and enhanced TCF1 expression 10-fold in murine T cells (Figures 2B and 2C). In human peripheral CD8^+^ T cells, MG-MIase increased CD62L^+^ CD45RO^+^ stem cell-like memory T cell (T_SCM_) by ∼2-fold and TCF1 expression by ∼3-fold (Figures S4B and S4C). To evaluate whether D-mannose from MG-MIase maintains stem-like T cells, we used an ACT model with B16-OVA melanoma and OVA-specific T cells cultured *ex vivo* with or without MG-MIase. Compared with MG controls, MG-MIase treatment enhanced stem-like features during *ex vivo* expansion and allowed ACT cells to retain stemness after transfer. In contrast, MG-treated cells lost stemness *in vivo*, indicating that continuous D-mannose production at the tumor site sustains the stem-like phenotype (Figure S4D). To confirm that the stem-like phenotype depended on D-mannose production, we next tested the requirement for D-fructose as a substrate. In the absence of D-fructose as a substrate, MG-MIase failed to generate D-mannose and could not induce stem-like properties in either murine or human T cells, supporting a causal link between D-mannose production and T cell stemness (Figures 2B and 2C; Figure S4B). Additionally, MG-MIase treatment reduced LAG-3^+^ cells from 72.73% to 48.35%, PD-1^+^ cells from 84.35% to 51.58%, and TIM-3^+^ cells from 81.9% to 53.98% (all *p* < 0.001) (Figure 2D) and lowered exhaustion levels in human T cells (Figures S4E and S4F). Functionally, MG-MIase increased IFN-γ by ∼1.2-fold, TNF-α by∼1.89-fold, and IL-2 by ∼2.1-fold compared with MG (all *p* < 0.001) (Figures 2E, S4G, and S4H). In cytotoxic assays, MG-MIase treatment increased OT-1-mediated lysis of OVA^+^ tumor cells by approximately 2- to 3-fold compared with MG under hypoxia (Figure 2F), and it similarly enhanced the cytotoxicity of human CAR-T cells (Figure S4I).

To mechanistically link D-mannose production to T cell stemness and function, we analyzed the transcriptomes of T cells treated with MG, MG-MIase, or D-mannose. Compared with MG-treated T cells, both MG-MIase- and D-mannose-treated T cells showed upregulation of stemness-related genes (Figure 2G). Both conditions upregulated stemness-associated genes and shifted metabolism toward reduced glycolysis and increased oxidative phosphorylation (Figures 2H and 2I). Importantly, Wnt signaling activity was significantly enhanced in MG-MIase-treated T cells. β-catenin, a key regulator of Tcf7/Lef1 expression, showed markedly increased O-GlcNAcylation, suggesting that D-mannose stabilizes β-catenin through O-GlcNAcylation, thereby reinforcing Wnt-β-catenin signaling and promoting stem-like differentiation (Figure 2J).

Collectively, these observations indicate that MG-MIase-derived D-mannose drives T cells toward a stem-like phenotype, reduces exhaustion, and enhances effector function, likely through metabolic reprogramming and Wnt/β-catenin signaling.

### Antitumor efficacy of MG-MIase *in vivo*

**Figure 3:**
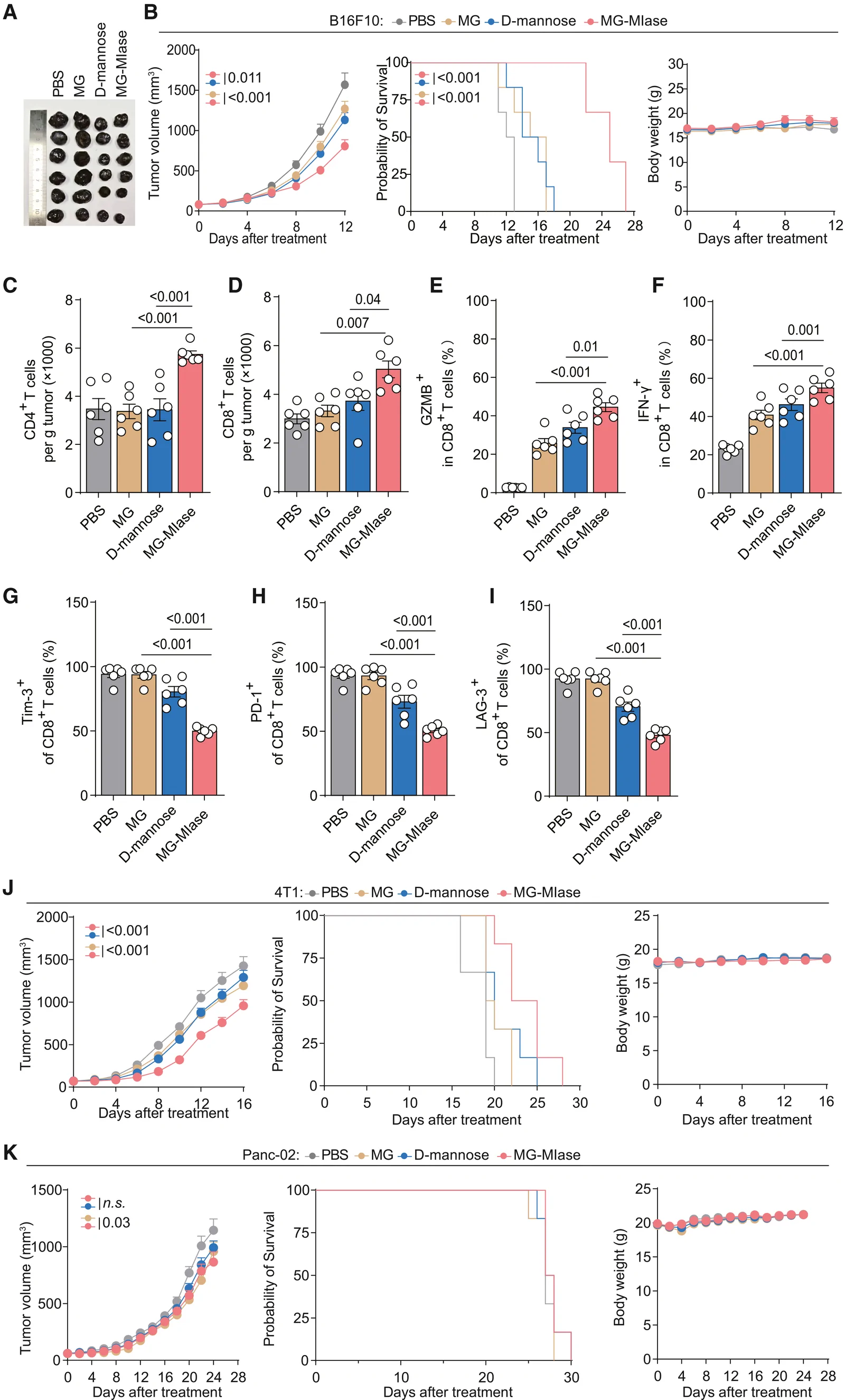
*In vivo* antitumor efficacy of MG-MIase (A) Images of B16F10 tumors from C57/BL6 mice (*n* = 6) treated with PBS, MG, D-mannose, or MG-MIase. (B) Tumor growth curves (left), body weight curves (middle), and survival curves (right) of B16F10-bearing mice after the indicated treatments. (C–F) PBS, MG, D-mannose, or MG-MIase was injected into B16F10 tumors, and tumor immune infiltrates were analyzed by flow cytometry after 5 days. (C) Counts of CD4^+^ T cells in tumors. (D) Counts of CD8^+^ T cells in tumors. (E) Frequencies of granzyme B^+^ CD8^+^ T cells in tumors. (F) Frequencies of IFN-γ^+^ CD8^+^ T cells in tumors. (G–I) Frequencies of exhaustion markers TIM-3 (G), PD-1 (H), and LAG-3 (I) on CD8^+^ T cells in tumors. (J) Tumor growth curves, body weight changes, and survival curves of mice after treatment in the 4T1 tumor model. (K) Tumor growth curves, body weight changes, and survival curves of mice after treatment in the Panc-02 tumor model. *p* values were calculated using one-way ANOVA with Tukey’s multiple comparison test (C–I) and two-way ANOVA following Fisher’s LSD test for multiple comparisons (B, L, and N). Data are mean ± SEM. *n.s.*, not significant.

To determine whether MG-MIase elicits a clinically meaningful immune response in immunocompetent hosts, we first assessed its antitumor activity in C57BL/6 mice bearing B16F10 melanoma. Tumor-bearing mice were randomized to receive *i.t.* MG-MIase, and oral mannose (20%)^23^ significantly delayed tumor progression and prolonged overall survival compared with PBS- and MG-treated controls (Figures 3A and 3B). Notably, MG-MIase achieved the most pronounced tumor control, whereas MG alone conferred only a modest growth delay, likely due to non-specific immune activation (Figures 3A and 3B). To further investigate the immunomodulatory effects of MG-MIase, we quantified cytokine levels in both tumor and serum samples by ELISA. Treatment with MG-MIase significantly elevated proinflammatory cytokines, including IFN-γ, IL-1β, and IL-6, while notably reducing levels of the anti-inflammatory cytokine IL-10 (Figures S5A and S5B). These findings suggest that MG-MIase facilitates the conversion of the TME from an immunologically “cold” to a “hot” state. Flow cytometric analysis performed 72 h post treatment revealed that MG-MIase enhanced intratumor infiltration of CD4^+^ (1.02-fold) and CD8^+^ (1.52-fold) T cell infiltration (Figures 3C and 3D). Notably, CD8^+^ T cells showed elevated granzyme B (1.02-fold) and IFN-γ (1.52-fold) expression (Figures 3E and 3F), whereas the proportions of PD-1^+^, LAG-3^+^, and TIM-3^+^ T cells were reduced by almost 50% (Figures 3G–3I). Together, these data demonstrated that MG-MIase reshapes the TME *in situ* by enabling hypoxia-restricted D-mannose production, thereby achieving stronger and more sustained antitumor immunity than systemic mannose supplementation.

Furthermore, mouse body weight remained largely unchanged in all groups (Figure 3B). To further evaluate safety, blood biochemical analyses were performed on tumor-bearing mice treated with MG-MIase. The serum levels of alanine aminotransferase (ALT) and aspartate aminotransferase (AST), two markers of liver damage, remained within normal ranges (Figures S5C and S5D). Furthermore, hematoxylin and eosin (H&E) staining of major organs—including the heart, liver, spleen, lungs, and kidneys—following both *i.t.* and *i.v.* injection of MG-MIase revealed no tissue damage, supporting the safety of the proposed treatment regimen (Figures S5E and S5F). Encouraged by this efficacy, we next evaluated MG-MIase in two immunogenic “cold” tumor models: 4T1 breast carcinoma and Panc-02 pancreatic carcinoma. In both models, MG-MIase delayed tumor growth compared with PBS and MG controls, confirming that its metabolic-immune mechanism is not restricted to melanoma. However, the extent of growth inhibition was modest, and survival was not significantly extended (Figures 3J and 3K). Taken together, these findings demonstrate that MG-MIase reprograms the TME, yielding effective tumor control and improved survival in immunogenic models without inducing systemic toxicity. Nevertheless, its attenuated response in low-immunogenic tumors underscores the need for further optimization.

### Construction and characterization of MG-MIase@TMV

**Figure 4:**
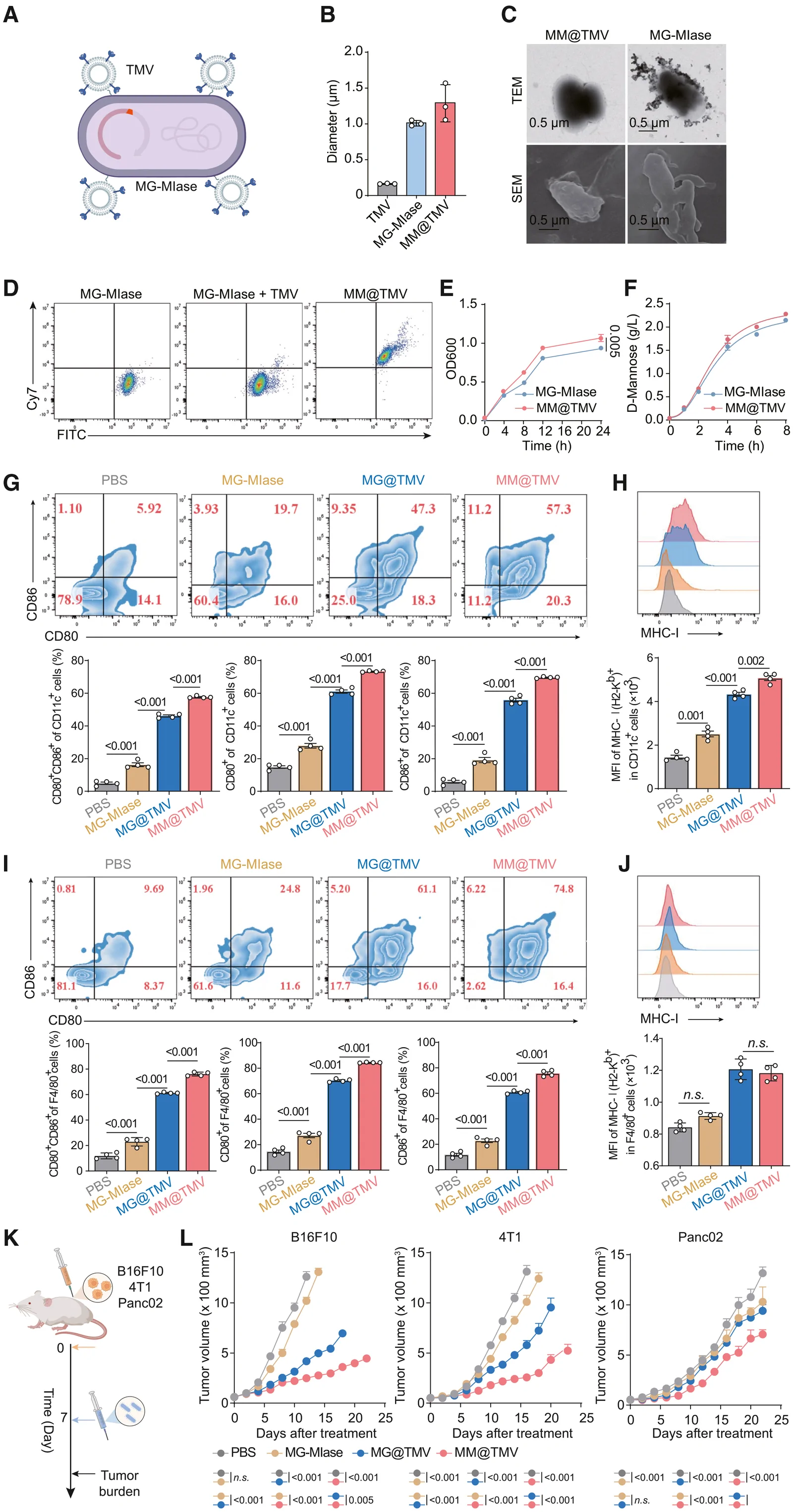
Preparation and characterization of MM@TMV and its *in vitro* antitumor activity (A) Schematic diagram of MM@TMV. (B) Particle size of TMV, MG-MIase, and MM@TMV. *n* = 3 samples/group. (C) TEM and SEM images of MG-MIase and MM@TMV. (D) Representative flow cytometry plots of fluorescence-labeled MM@TMV. (E) The growth activity of MG-MIase and MM@TMV, as indicated by OD600. *n* = 3 samples/group. (F) The catalytic ability of MM@TMV to convert fructose into mannose. *n* = 5 samples/group. (G) Representative flow cytometry plots and quantitative analysis of CD80^+^CD86^+^ expression in BMDCs after co-culture with PBS, MG-MIase, MG@TMV, or MM@TMV. *n* = 4 samples/group. (H) Representative histograms and quantitative analysis of MHC-Ⅰ expression in BMDCs after co-culture. *n* = 4 samples/group. (I) Representative flow cytometry plots and quantitative analysis of CD80^+^CD86^+^ expression in BMDMs after co-culture. *n* = 4 samples/group. (J) Representative histograms and quantitative analysis of MHC-Ⅰ expression in BMDMs after co-culture. *n* = 4 samples/group. (K) Scheme for the experimental protocols for antitumor evaluation of MM@TMV. (L) Tumor growth curves of mice after treatment in B16F10, 4T1, and Panc-02 tumor models. *n* = 5 samples/group. *p* values were calculated using one-way ANOVA with Tukey’s multiple comparison test (B and G–I) and two-way ANOVA following Fisher’s LSD test for multiple comparisons (E, F, and L). Data are mean ± SEM.

Although MG-MIase alone showed promising antitumor efficacy in melanoma models, it exhibited limited therapeutic effects in refractory tumors such as 4T1 and Panc-02, indicating that additional mechanisms are required to overcome immune resistance. We reasoned that, in these antigen-poor and immune-resistant tumors, metabolic modulation alone may be insufficient to induce robust T cell responses without concurrent antigenic stimulation. Therefore, to integrate metabolic reprogramming with enhanced antigen presentation, we functionalized MG-MIase with TMVs, which carry endogenous tumor antigens. TMVs were anchored onto MG-MIase using a dual-linking strategy. Stable covalent attachment to the bacterial surface was achieved through amide bond (-CONH-) formation between carboxyl groups on TMVs and primary amino groups on the bacterial outer membrane. In parallel, DSPE lipid insertion was employed on the TMV’s side to stabilize membrane association while retaining the capacity for dissociation during antigen-presenting cell uptake. Following conjugation, the reaction mixture was centrifuged at 8,000 rpm to selectively pellet the bacterial carriers, thereby removing unbound or self-associated TMVs and enriching the intended MG-MIase@TMV bacterial construct (Figure 4A). TMVs and enriching the intended MG-MIase@TMV construct (hereafter referred to as MM@TMV). In addition, MG conjugated with TMVs (MG@TMV) was prepared using the same method. As shown in Figure 4B, dynamic light scattering analysis revealed that the average particle sizes of the prepared TMVs, MG-MIase, and MM@TMV were 164.7 nm, 1,008.7 nm, and 1,287.3 nm, respectively. The presence of TMVs on the surface of MG-MIase was characterized by transmission electron microscopy (TEM) and scanning electron microscopy (SEM) images. Compared with the smooth surface morphology of untreated MG-MIase, spherical particles were clearly observed on the surface of TMV-modified MG-MIase, indicating that TMVs were successfully conjugated to the surface of the probiotic (Figure 4C). In addition, we labeled the engineered bacteria with fluorescein isothiocyanate (FITC) and the TMVs with near-infrared fluorescent dye Cyanine7 (Cy7). The successful conjugation of TMVs on the surface of MG-MIase was verified by detecting fluorescence from the engineered bacteria through flow cytometry. TMVs without DSPE-NHS modification did not bind nonspecifically to the bacterial surface (Figure 4D). Importantly, the surface conjugation of TMVs showed limited impact on bacterial viability, as the proliferation of MG-MIase was similar to that of unmodified bacteria (Figure 4E). Subsequently, we found that TMV modification did not diminish the D-mannose-synthesizing ability of MM@TMV (Figure 4F). We next examined whether TMV modification altered the *in vivo* colonization and clearance profile of the engineered bacteria. In tumor-bearing mice, CFU quantification showed that MM@TMV progressively accumulated and persisted within tumors, whereas bacterial burdens in the heart, liver, spleen, lungs, and kidneys declined to nearly undetectable levels by day 7 (Figure S6A). In parallel, healthy non-tumor-bearing mice displayed a similar systemic clearance from all examined organs, with no long-term bacterial reservoirs observed (Figure S6B). *In vivo* tracking of MM@TMV via fluorescence signals further confirmed this observation (Figures S6C and S6D). In addition, D-mannose levels remained elevated only in tumors after colonization, confirming sustained intratumoral mannose production by MM@TMV (Figure S6E).

To verify that TMV anchoring allows antigen uptake by APCs while preserving the intratumoral metabolic activity of MG-MIase, we co-incubated MM@TMV with bone-marrow-derived dendritic cells (BMDCs). In our design, TMVs attached to the bacterial surface through DSPE insertion, so that once MM@TMV enters the tumor, APCs preferentially internalize the TMVs rather than engulfing the entire bacterial carriers. Consistent with this, mannose concentrations remained high in the medium after DC co-culture, confirming that MM@TMV retains enzymatic activity even following APC-mediated TMV uptake (Figures S7A–S7C).

### Investigation of the immune activation induced by MM@TMV system

To investigate the immune activation induced by MM@TMV, bone marrow-derived dendritic cells (BMDCs) were co-incubated for 24 h and assessed for maturation markers and antigen-presenting function. The expression of the activation markers CD80 and CD86 in BMDCs was significantly upregulated in both the MG@TMV and MM@TMV groups. In the MM@TMV group, the BMDCs’ maturation rate reached 58.7%, which was 3.9-fold and 1.2-fold higher than those in the MG-MIase and MG@TMV groups, respectively (Figure 4G). Furthermore, MM@TMV treatment increased the antigen-presenting ability of BMDCs, as indicated by a 2-fold increase in the proportion of MHC-I (H2-K^b+^) cells compared with the control group, which was involved in antigen presentation to T cells expressing CD3/TCR and CD8^+^ T cells (Figure 4H). These results demonstrate that the engineered probiotic-based tumor antigen delivery system, MM@TMV, significantly induces BMDC maturation and enhances antigen-delivery efficiency. A similar trend was observed in BMDMs, which exhibited increased proportions of CD80^+^CD86^+^ and MHC-I (H2-K^b+^) cells, indicating polarization toward a proinflammatory M1 phenotype in response to TMV stimulation (Figures 4I and 4J).

We next sought to provide further evidence that MM@TMV not only promotes maturation of APCs but also enhances their antigen-presenting capacity *in vivo* by upregulating MHC expression. Tumor tissues were collected on the fifth day after treatment to evaluate the immune responses triggered by MM@TMV (Figure S8A). Compared with the other three groups, the MM@TMV group showed a significantly higher proportion of CD80^+^CD86^+^ dendritic cells in the tumor, along with elevated CD86 expression in macrophages. The M1/M2 ratio was increased compared with the other groups, indicating that MM@TMV effectively repolarized M2-type macrophages into the proinflammatory M1 phenotype *in vivo* (Figures S8B and S8C).

Next, we further investigated the activation of intratumoral T cells. Notably, compared with the other groups, the MM@TMV-treated group exhibited a significantly increased proportion of CD8^+^ T cells co-expressing CD69 in the tumor tissues of mice, a marker of T cell activation (Figure S8D). Meanwhile, the proportion of IFN-γ^+^ CD8T^+^ T cells increased by 2.7-fold and granzyme B^+^ CD8T^+^ cells by 2.6-fold in the MM@TMV group relative to MG-MIase (Figures S8E and S8F). The increased infiltration of cytotoxic T lymphocytes (CTLs) into the tumor tissues—typically regarded as the primary effector cells responsible for tumor cell killing—further demonstrated the effectiveness of MM@TMV in enhancing T cell-mediated immune responses.

Furthermore, to test whether MM@TMV can overcome the poor responsiveness of refractory “cold” tumors, we evaluated its therapeutic efficacy in mouse models of B16F10 melanoma, as well as low-immunogenic 4T1 breast cancer and Panc-02 pancreatic cancer. Building on our aim to boost intratumor T cell activation via enhanced antigen-delivery, tumor-bearing mice were divided into four groups and received *i.t.* injections on day 0: PBS group, MG-MIase group, MG@TMV group, and MM@TMV group (Figure 4K). Strikingly, MM@TMV treatment produced notable therapeutic effects across all three models, producing the smallest average tumor volume and the highest survival rates compared with control groups (Figure 4L). Together, these results demonstrated that our strategy indeed achieves robust antitumor efficacy against otherwise refractory tumors.

Finally, given the potential safety concerns associated with *i.v.* bacterial administration, we conducted a comprehensive safety evaluation in Panc-02 tumor-bearing mice. Throughout the treatment period, physiological monitoring was continuously performed, including behavioral scoring and routine measurements of body temperature, body weight, blood pressure, and heart rate. On day 16, blood samples were collected for further analysis (Figure S9A). Across all treatment groups, no significant differences were observed in body temperature (°C), body weight (g), mean blood pressure (mm Hg), or heart rate (bpm), confirming the absence of systemic toxicity (Figures S9B–S9E). Completed blood cell count analysis further revealed no significant difference in white blood cells, lymphocytes, monocytes, neutrophils, red blood cells, or platelets among groups (all *p* > 0.05), demonstrating that MG-MIase and MM@TMV treatment did not induce systemic hematologic abnormalities (Figure S9F). Collectively, these results support a favorable safety profile, indicating an absence of systemic hematologic toxicity.

### MM@TMV enhances the tumor-targeting ability and therapeutic efficacy of TCS9R-T cells

**Figure 5:**
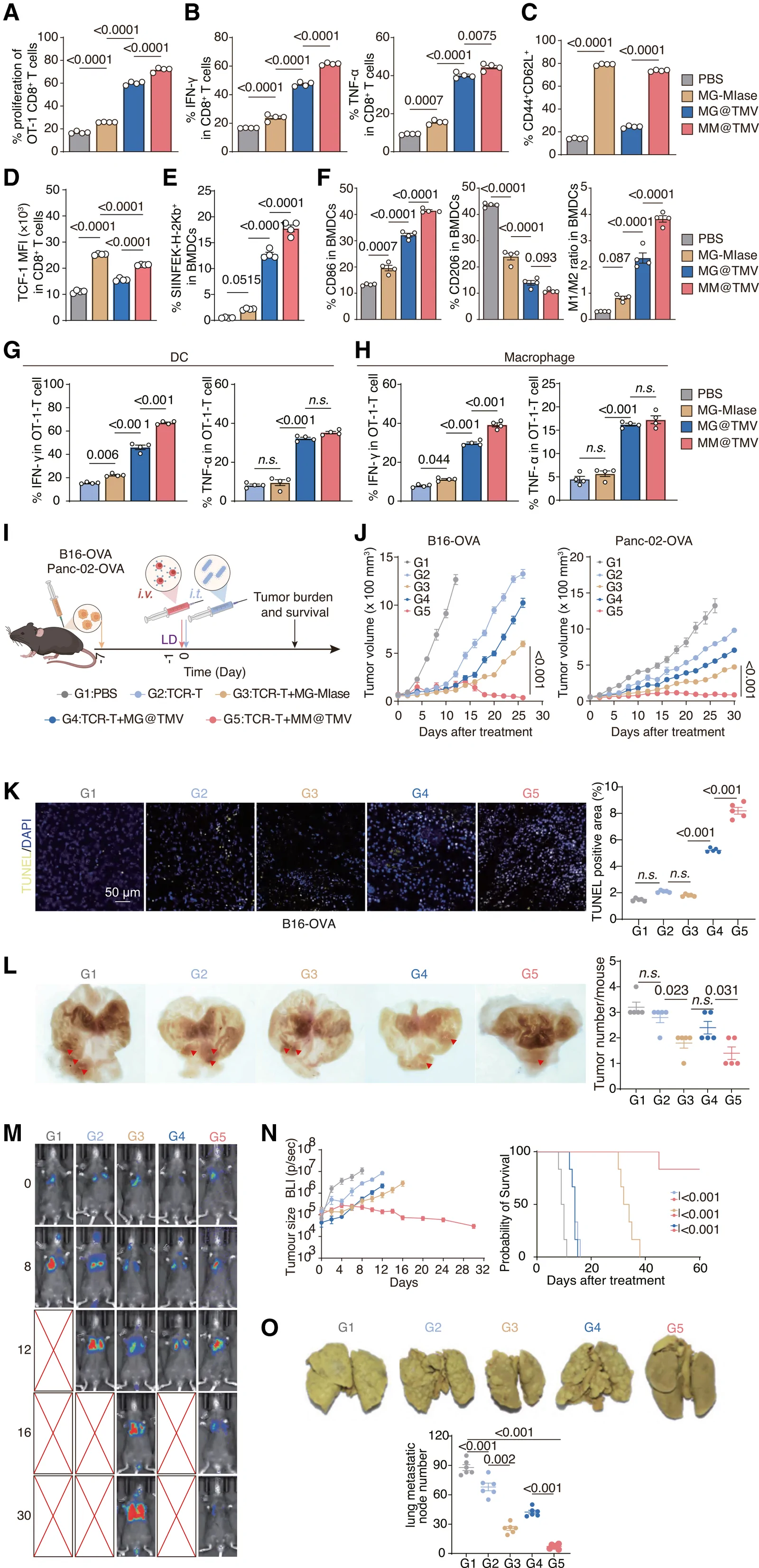
MM@TMV significantly enhanced the antitumor efficacy of TCR-T cells (A) Quantitative analysis of the proliferation rate of CD8^+^T cells in indicated conditions at day 5. *n* = 4 samples/group. (B) Quantitative analysis of intracellular IFN-γ and TNF-α in CD8^+^ T cells by flow cytometry. *n* = 4 samples/group. (C) Quantitative analysis of CD44^+^CD62L^+^ expression in CD8^+^ T cells. *n* = 4 samples/group. (D) Quantitative analysis of the MFI of TCF-1 in CD8^+^ T cells under indicated conditions. *n* = 4 samples/group. (E) Quantitative analysis of SIINFEKL-H-2Kb presentation in BMDCs. *n* = 4 samples/group. (F) Quantitative analysis of CD86^+^, CD206^+^ expression and M1/M2 ratio in BMDMs. *n* = 4 samples/group. (G) Quantitative analysis of the percentage of IFNγ^+^ and TNF-α^+^ cells in OT-1 CD8^+^ T cells after co-culture with DCs. *n* = 4 samples/group. (H) Quantitative analysis of the percentage of IFN-γ and TNF-α cells in OT-1 CD8^+^ T cells after co-culture with macrophages. *n* = 4 samples/group. (I) Scheme for the experimental protocols of enhanced antitumor evaluation of TCR-T cells by MM@TMV. *n* = 5 samples/group. (J) Tumor growth curves and survival of mice after treatment in B16-OVA (left) and Panc-02-OVA (right) tumor models. (K) TUNEL staining of tumor sections. Apoptotic cells are shown in yellow. Scale bar, 100 μm. *n* = 5 samples/group. (L) Representative images of the stomach (left) from orthotopic gastric tumor-bearing mice demonstrate the presence of adenomas in the antral region. The quantitative analysis of tumor number is shown on the right. (M and N) Representative bioluminescence images (M), quantification of bioluminescence intensity reflecting tumor burden (N, left), and survival curves of mice undergoing treatment (N, right). (O) Representative lung images in the Panc-02-OVA metastasis model (top). Number of the lung metastatic nodules (bottom). *n* = 6 samples/group. *p* values were calculated using one-way ANOVA with Tukey’s multiple comparison test (A–H, K–L, and O) and two-way ANOVA following Fisher’s LSD test for multiple comparisons (J and N). Data are mean ± SEM. *n.s.*, not significant.

Adoptive T cell therapies such as CAR-T cells have demonstrated considerable potential in targeting tumor-specific antigens and mediating tumor regression. To assess the potential of MM@TMV in enhancing ACT, we first evaluated the antitumor activity of TCR-T alone in syngeneic mouse models. CD8^+^ T cells specific for OVA_257_–_264_ epitope were isolated from OT-1 mice and co-cultured with MM@TMV for 5 days. Flow cytometry confirmed efficient activation of TCR-T cells by TMV-presented MHC-I–OVA_257_–_264_ complexes (Figure 5A). MM@TMV stimulation led to a quantifiable increase in IFN-γ^+^ and TNF-α^+^ TCR-T cells relative to MG-MIase and MG@TMV (Figure 5B; Figure S10A), indicating stronger effector licensing under antigen-coupled mannose delivery. Strikingly, after treatment with MM@TMV, TCR-T cells maintained a high proportion of CD62L^+^CD44^+^ and TCF1 phenotypes (Figures 5C and 5D; Figures S10B and S10C), indicating that MM@TMV activates TCR-T cells while preserving their stemness and preventing exhaustion caused by overstimulation.

To examine the antigen presentation after MM@TMV treatment, BMDCs or BMDMs were incubated with MM@TMV. MM@TMV treatment enhanced the antigen-presenting ability of BMDCs, as evidenced by a 9-fold increase in the level of MHC-I–OVA_257–264_ complexes compared with the MG-MIase group (Figure 5E; Figure S10D). Additionally, MM@TMV treatment increased the number of M1-like macrophages (Figure 5F). Furthermore, co-incubation of MM@TMV-treated BMDCs and BMDMs with naive OT-1 CD8^+^ T cells enhanced T cell effector functions, demonstrating robust antigen cross-presentation and strong immune activation of T cells by antigen-presenting cells (Figures 5G and 5H; Figures S10E and S10F) demonstrating robust cross-presentation and strong APCs-to-T immune activation.

Subsequently, we combined MM@TMV with TCR-T cells in the B16-OVA and Panc-02-OVA tumor models to evaluate their therapeutic efficacy and assess whether MM@TMV could enhance the antitumor effect of adoptively transferred T cells *in vivo*, following the experimental timelines outlined in Figure 5I. TCR-T cells alone inhibited tumor growth, indicating their ability to specifically recognize and kill OVA-expressing tumors. In contrast, MG-MIase and MG@TMV temporarily enhanced the antitumor effect of TCR-T cells but failed to induce sustained tumor regression. However, adoptive transfer of TCR-T cells combined with MM@TMV induced complete tumor regression and durable cures in most treated mice (Figure 5J). TUNEL staining showed a higher abundance of apoptotic tumor cells in the MM@TMV plus TCR-T cell group relative to all controls, consistent with the observed antitumor effect (Figure 5K). We then extended this strategy to the poorly immunogenic Panc-02-OVA tumors, which are poorly immunogenic, as previous treatment with MG-MIase alone failed to effectively inhibit tumor growth (Figures 3E and 3F). The results showed that, compared with MG-MIase, MM@TMV further enhanced the antitumor efficacy of TCR-T cells, leading to marked tumor regression in mice (complete remission, CR = 6/6), demonstrating its potent therapeutic effect (Figure 5J). After the mice were cured, MM@TMV was completely cleared from the body, further demonstrating the safety of this therapy (Figures S10G and S10H).

We also established an orthotopic gastric cancer (GC) allograft mouse model by injecting YTN16-OVA cells into the serosal layer of the stomach.^38^ Tumor formation was confirmed by gross examination and H&E staining of the stomach (Figure S10I). Mice were then treated with MM@TMV combined with TCR-T cells. Compared with other groups, the combination of MM@TMV and TCR-T cells significantly reduced the tumor burden, as evidenced by a decreased tumor number (Figure 5L). Next, we evaluated whether the combination of MM@TMV and TCR-T cells could control metastatic cancer. To establish a metastasis tumor model, C57BL/6 mice were intravenously injected with Panc-02-OVA-luc cells to establish a lung metastasis model. After metastatic nodules formed in the lungs, the mice were subjected to sublethal lymphodepletion and then received adoptive transfer of TCR-T cells alone or in combination with MG-MIase, MG@TMV, or MM@TMV. Tumor progression was monitored by bioluminescence imaging. Notably, TCR-T cells combined with MM@TMV exhibited superior antimetastatic activity, achieving 100% durable cures in all treated mice, whereas TCR-T cells alone or in combination with either MG-MIase or MG@TMV provided only modestly controlled tumor burden (Figures 5M and 5N). Consistently, the combination therapy also significantly prolonged overall survival compared with the other groups (Figure 5N). Picric acid-based counting showed that MM@TMV combined with TCR-T cells treatment resulted in the fewest number of lung metastatic nodules compared with other treatments (Figure 5O). These results suggested strong antimetastatic effects of MM@TMV combined with TCR-T cell therapy for treating systemic metastasis tumors.

### MM@TMV therapy ameliorates the immunosuppressive microenvironment and promotes adoptive T cell therapy

**Figure 6:**
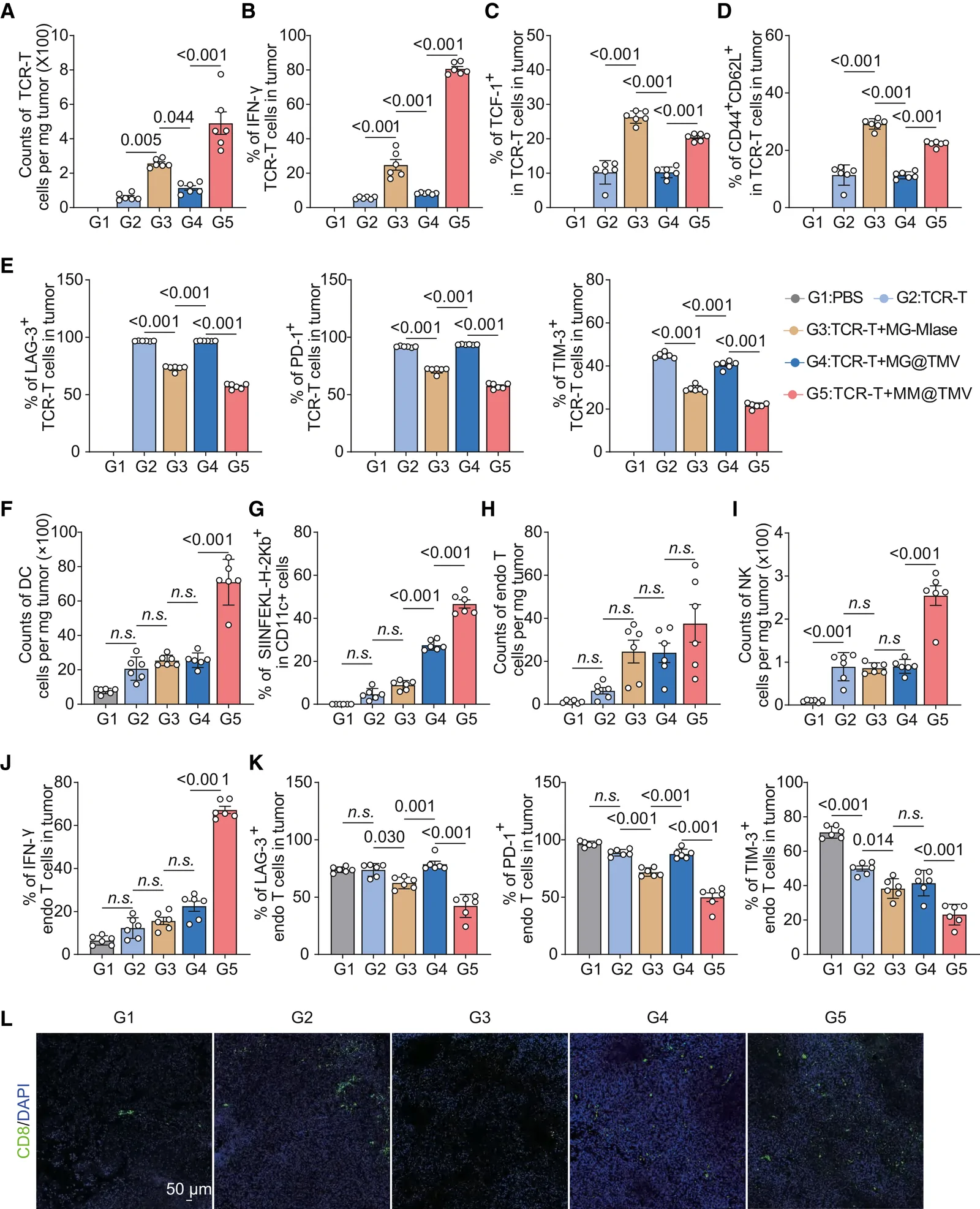
Mechanistic study of the significant enhancement of TCR-T cells antitumor efficacy by MM@TMV *in vivo* (A) Quantitative analysis of the number of TCR-T cells per mg tumor. (B) Quantitative analysis of intracellular IFN-γ in TCR-T cells. (C) Quantitative analysis of the percentage of TCF-1^+^ cells in TCR-T cells. (D) Quantitative analysis of the percentage of CD44^+^CD62L^+^ cells in TCR-T cells. (E) Quantitative analysis of the expression of exhaustion markers TIM-3, PD-1, and LAG-3 in TCR-T cells in tumors. (F) Quantitative analysis of the number of DCs per mg tumor. (G) Quantitative analysis of SIINFEKL-H-2Kb presentation in DCs in tumor. (H) Quantitative analysis of the number of endogenous T cells per mg tumor. (I) Quantitative analysis of the number of NK cells per mg tumor. (J) Quantitative analysis of intracellular IFN-γ in endogenous T cells. (K) Quantitative analysis of the expression of exhaustion markers TIM-3, PD-1, and LAG-3 in endogenous T cells in tumors. (L) CD8 immunofluorescence staining of tumor sections (green). Scale bar, 100 μm. *n* = 5 samples/group. *p* values were calculated using one-way ANOVA with Tukey’s multiple comparison test. Data are mean ± SEM. All experiments, *n* = 6 samples/group. *n.s.*, not significant.

To further comprehensively evaluate the systemic antitumor response, we analyzed immune cells in the Panc-02-OVA tumor using flow cytometry (Figure S11A). Compared with the TCR-T cell group, MM@TMV-treated TCR-T cells showed approximately a 5.3-fold increase in cell counts within the tumor tissue, which was 1.8-fold and 4.75-fold higher than those treated with MG-MIase and MG@TMV, respectively (Figure 6A). Meanwhile, IFN-γ secretion by TCR-T cells was increased by 14-fold in the MM@TMV group relative to TCR-T alone and by 3.2-fold relative to MG-MIase (Figure 6B; Figure S11B), supporting a direct enhancement of adoptive T cell responses. Moreover, TCR-T cells in the MM@TMV treatment group were still able to maintain higher levels of TCF1 in tumors (Figure 6C; Figure S11C), along with a higher proportion of CD44^+^CD62L^+^ cells within the tumor tissues (Figure 6D; Figures S11D and S11E). In addition, inhibitory receptors were reduced in MM@TMV-treated TCR-T cells, with PD-1, TIM-3, and LAG-3 levels decreased by approximately 60%–80% relative to TCR-T alone (Figure 6E; Figures S11F and S11G). These results indicated that D-mannose synthesized by MM@TMV could regulate TCR-T cells to maintain an optimal metabolic state, prevent exhaustion caused by excessive activation, and thereby provide sustained therapeutic effects.

Next, we investigated the effects of this therapy on the intratumoral immune system. There was a significant increase in the number of DCs within the tumor and a substantial increase in SIINFEKL presentation by DCs (Figures 6F and 6G; Figures S11H and S11I). This further facilitated cross-presentation of the SIINFEKL-H-2Kb complex to T cells in the tumor, thereby reactivating their antitumor functions. As a result, the numbers of endogenous T cells and NK cells in the tumor tissue also increased significantly (Figures 6H and 6I). Moreover, endogenous CD8^+^ T cells showed elevated IFN-γ production, increasing by 5.4-fold compared with TCR-T alone group (Figure 6J; Figure S11J). In addition, endogenous T cells exhibited reduced exhaustion (Figure 6K; Figure S11K). Consistent with the flow cytometry results, immunofluorescence staining further confirmed that CD8^+^ T cells showed significantly enhanced intratumoral infiltration in the MM@TMV-treated group (Figure 6L). Collectively, these findings indicated that MM@TMV treatment not only reversed the immunosuppressive TME and enhanced the antitumor functions of both adoptively transferred and endogenous immune cells, but it also improved T cell metabolic states through D-mannose synthesis, maintaining a stem-like phenotype.

To directly examine whether MM@TMV treatment alters the metabolic state of adoptively transferred T cells within tumors, TCR-T cells were isolated from B16F10-OVA tumor tissues (Figure S12A) and subjected to Seahorse mitochondrial stress testing. Compared with TCR-T cells from control groups, MM@TMV-treated TCR-T cells exhibited significantly higher oxygen consumption rate (OCR) and increased spare respiratory capacity, indicating enhanced oxidative phosphorylation and greater metabolic fitness (Figures S12B and S12C). In contrast, extracellular acidification rates (ECARs) were reduced, suggesting a lower reliance on glycolytic metabolism (Figure S12D). This metabolic profile is characteristic of less differentiated memory-like CD8^+^ T cells, including TCM/TSCM subsets, and indicates improved bioenergetic adaptability in MM@TMV-treated TCR-T cells.

Subsequently, to evaluate the specific contribution of TMV-mediated antigen presentation to the overall therapeutic effect, we generated membrane vesicles derived from an unrelated cell line and lacking the relevant antigen (nTMVs from 4T1 cells) and constructed MM@nTMV (Figure S12A). *In vivo* experiments showed that engineered bacteria loaded with non-antigen-bearing membrane vesicles did not enhance the antitumor activity of TCR-T cells (Figure S12F). Further analysis of tumor-infiltrating DCs revealed that, compared with antigen-bearing TMVs, nTMVs failed to effectively activate DCs (18.76% ± 2.16% vs. 42.88% ± 4.25%) or promote DC-mediated presentation of SIINFEKL to drive TCR-T cell antitumor responses (13.56% ± 3.58% vs. 48.96% ± 3.94%). In addition, nTMVs exhibited only limited ability to activate macrophages and support antigen presentation (Figures S12G–S12J). These results indicate that antigen-bearing TMVs can be efficiently internalized by APCs and processed for antigen presentation, thereby eliciting a strong and antigen-specific T cell response.

### MM@TMV enhances CAR-T cell-mediated immunotherapy in a humanized PDX model

**Figure 7:**
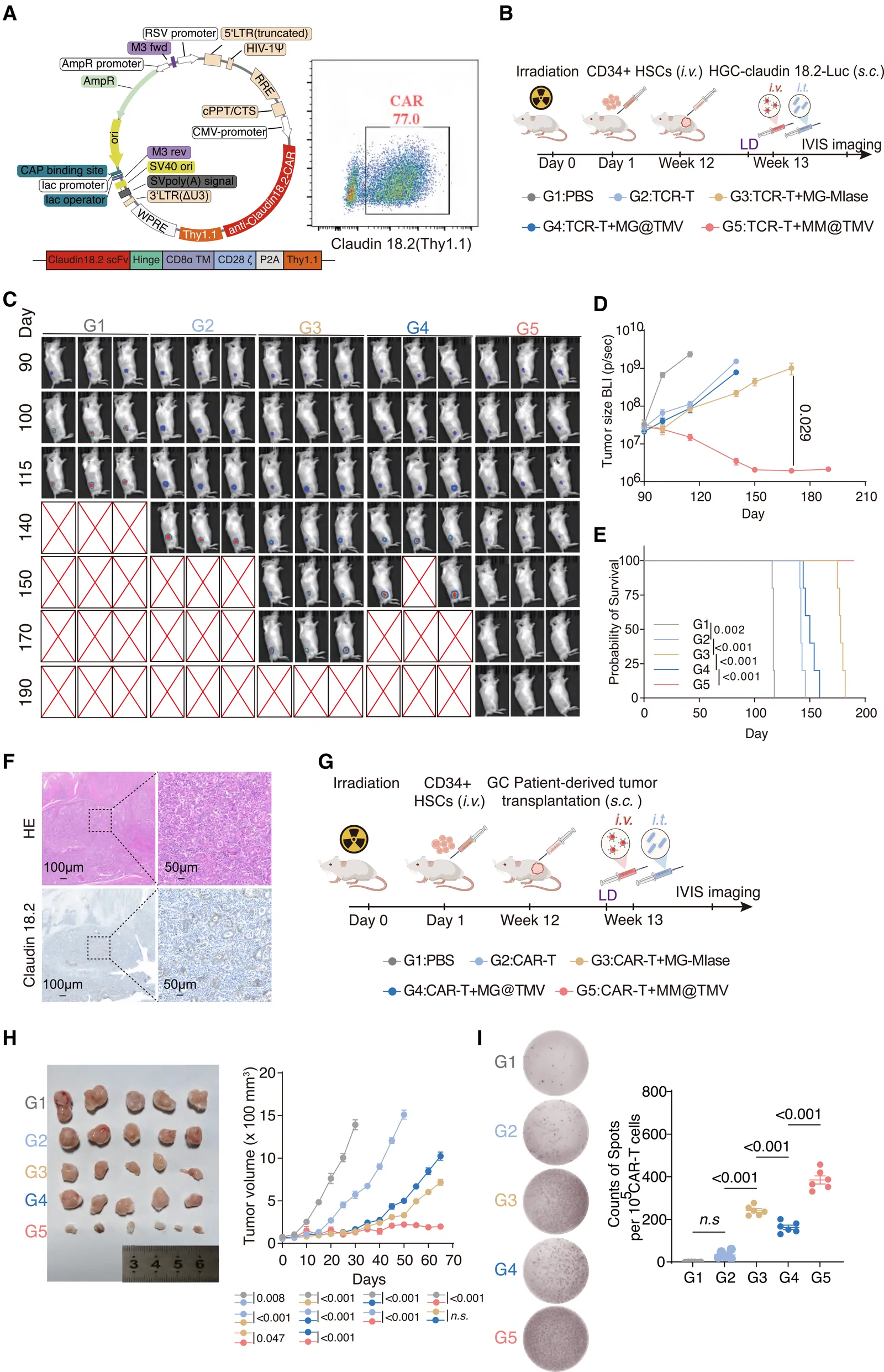
Therapeutic efficacy of MM@TMV with CAR-T cells against humanized PDX models (A) Schematic of the CAR construct (left) and flow cytometric analysis of CAR expression in T cells (right). (B) Experimental process. (C) Representative bioluminescence images and (D) Quantification of bioluminescence intensity. (E) Survival curves. (F) Representative H&E and Claudin18.2 IHC images from the same gastric cancer patient, showing Claudin18.2 expression in invasive tumor glands. (G) Schematic illustration of the experimental design for the humanized PDX models. (H) Tumor growth curves. (I) Representative ELISPOT images and quantification the number of the lung metastatic nodules. All experiments, *n* = 5 samples/group. *p* values were calculated using one-way ANOVA with Tukey’s multiple comparison test and two-way ANOVA following Fisher’s LSD test for multiple comparisons. Data are mean ± SEM. *n.s.*, not significant.

Given the promising preclinical results of MM@TMV in enhancing murine TCR-T cell responses, we then sought to evaluate its ability to potentiate CAR-T cell therapy in a humanized mouse model. Claudin18.2, a tight junction protein highly expressed in GC, has recently emerged as a clinically actionable target. Notably, Claudin18.2-specific CAR-T cells have shown encouraging therapeutic outcomes in early-phase clinical trials for gastric and gastroesophageal cancers.^39^ To investigate whether MM@TMV could augment the efficacy of Claudin18.2-targeting CAR-T cells, we selected the GC cell line HGC-27, which exhibits high expression of Claudin18.2,^40^ and prepared MM@TMV carrying the Claudin18.2 antigen, thereby creating an MM@TMV system capable of activating Claudin18.2-specific CAR-T cells. Subsequently, we successfully generated Claudin18.2 CAR-T cells. In brief, a tandem construct was used to encode the second-generation anti-Claudin18.2 CAR and Thy1.1 as a reporter gene, linked by a cleavable 2A peptide sequence. Flow cytometry analysis revealed that approximately 70% of the successfully constructed CAR-T cells expressed anti-Claudin18.2 CAR (Figure 7A; Table S2).

Because the MM@TMV system requires the coordinated action of multiple immune cell types, we constructed an immunocompetent model. After human hematopoietic stem cells (HSCs) were transplanted into irradiated NCG mice, macrophage reconstitution was assessed by flow cytometry before treatment allocation. Human macrophages, defined as hCD45^+^CD11b^+^hCD14^+^ cells, accounted for approximately 9% of the reconstituted human immune cells (Figure 7B; Figure S13A). A humanized HGC-27-Claudin18.2 model was successfully established. MM@TMV in combination with CAR-T cells elicited the most pronounced antitumor effect, driving tumor volumes to near-baseline levels and surpassing CAR-T alone as well as the MG-MIase- and MG@TMV-based combinations (Figures 7C–7E).

To further validate the clinical potential of MM@TMV therapy, we established a humanized GC patient-derived xenograft (PDX) model derived from a GC patient (Figures 7F and 7G). In this humanized PDX model, MM@TMV treatment also increased human M1 like macrophages, decreased human M2 like macrophages, and elevated the M1/M2 ratio within tumor (Figures S13C–S13F). The results demonstrated that MM@TMV combined with CAR-T cells treatment significantly inhibited tumor growth in this humanized model (Figure 7H).

We next evaluated Claudin18.2 expression after therapy to assess antigen escape. CAR-T monotherapy increased the proportion of Claudin18.2-negative (Claudin18.2^−^) tumor cells from 46.7% in the PBS group to 80.6%, without significantly reducing their absolute number, indicating relative enrichment of antigen loss variants. In contrast, MM@TMV combined with CAR-T did not increase the proportion of Claudin18.2^−^ cells relative to the PBS group and markedly reduced their absolute number compared with the other treatment groups, suggesting suppression of antigen-negative outgrowth (Figures S14A–S14C). To further explore whether this combination strategy could help overcome antigen heterogeneity, we established a mixed tumor model using Panc02-hCD19-OVA and Panc02-OVA cells and treated mice with hCD19-specific CAR-T cells. Tetramer staining showed that MM@TMV combined with CAR-T treatment increased the frequency of SIIN specific CD8^+^ T cells within tumors, suggesting enhanced antigen spreading and broader endogenous CD8^+^ T cell activation (Figures S14D and S14E). CAR-T cells isolated from tumors were co-cultured with tumor cells, and enzyme-linked immunosorbent spot (ELISPOT) analysis of IFN-γ production revealed that CAR-T cells from the MM@TMV treatment group exhibited a markedly stronger immune response against tumor cells (Figure 7I). These findings highlight the therapeutic efficacy of MM@TMV in clinically relevant models and underscore its potential for future clinical application.

## Discussion

Metabolic and microenvironmental modulation has emerged as an important direction for improving adoptive T cell therapy in solid tumors.^12^^,^^41^ A number of strategies have been explored, including engineering ACT cells to enhance persistence and function, improving tumor infiltration, reducing exhaustion, and reshaping the immunosuppressive TME.^5^^,^^12^^,^^41^^,^^42^^,^^43^ However, many of these approaches address only one dimension of the problem and do not fully resolve the coupled barriers of metabolic restriction, inadequate antigen presentation, and progressive T cell dysfunction within solid tumors.^44^ Here, we developed MM@TMV, a programmable hybrid platform that integrates hypoxia-responsive engineered bacteria with TMVs to enable localized mannose generation together with enhanced tumor antigen delivery and antigen-presenting cell activation. This coordinated design produced sustained antitumor activity across multiple syngeneic, refractory, and humanized models, supporting a therapeutic strategy that couples metabolic rewiring with immune reinforcement to improve ACT in solid tumors.

Mannose, after cellular uptake, can be incorporated into intracellular pathways linked to carbohydrate metabolism and glycosylation, thereby influencing glycolysis, oxidative phosphorylation, and downstream signaling.^20^^,^^45^^,^^46^ In multiple disease processes, mannose has been implicated not only as a metabolic intermediate but also as a regulator of cell function through glycosylation-associated control of protein activity. Recent studies further showed that D-mannose promotes OGT-mediated O-GlcNAcylation of β-catenin, thereby preserving Tcf7 expression and stemness-related programs in T cells.^23^ In the present study, we translated this biology into a tumor-localized therapeutic strategy by using hypoxia-responsive engineered bacteria to generate mannose directly within the TME. We showed that under hypoxic conditions, MG-MIase converts TME-abundant D-fructose into D-mannose *in situ*. Consistent with this, local mannose generation shifted T cell metabolism away from glycolysis and toward oxidative phosphorylation, increased β-catenin O-GlcNAcylation, promoted marked upregulation of TCF1, expanded CD44-positive CD62L-positive stem-like memory T cells, and reduced exhaustion markers including PD1, TIM3, and LAG3. By generating mannose directly within the tumor bed, our engineered system may overcome the limited tumor accumulation and rapid clearance that are likely to constrain systemic mannose supplementation. Collectively, our findings support the feasibility of engaging a known immunometabolic program *in situ* to improve ACT efficacy in solid tumors.

A key translational consideration for the MG-MIase module is substrate availability, because its therapeutic activity depends on local conversion of fructose to mannose. In modern diets, systemic fructose exposure is common, and studies in several tumor models, including melanoma, breast cancer, and cervical cancer, suggest that some tumor cells may not readily utilize fructose directly because of limited ketohexokinase-C expression.^47^ This raises the possibility that fructose may remain sufficiently available in at least some tumors to support intratumoral mannose generation. In our study, although intratumoral fructose levels were not directly measured, MG-MIase effectively increased local mannose abundance and enhanced T cell function in multiple models, including melanoma and pancreatic tumors, indicating that substrate availability was sufficient in these settings. However, we did not systematically evaluate fructose abundance across different tumor types, nor did we compare how variation in local fructose availability influences mannose generation and T cell benefit. These issues warrant further investigation. In addition, because fructose has been reported to promote tumor growth in some contexts,^47^^,^^48^^,^^49^ the potential benefit and risk of external fructose supplementation must be carefully evaluated in dedicated animal studies before such an approach can be considered for clinical use.

While metabolic intervention through *in situ* mannose generation delayed tumor progression in melanoma models, its efficacy was attenuated in poorly immunogenic tumors such as 4T1 and Panc-02. This finding indicates that metabolic support alone is not sufficient to overcome profound immune evasion. The incorporation of TMVs effectively addressed this critical limitation. We showed that TMVs anchored to the bacterial surface efficiently delivered a complete repertoire of TAAs to APCs. This process markedly promoted the maturation of BMDCs and macrophages, as reflected by the upregulation of CD80 and CD86 and the enhancement of MHC-I-mediated cross-presentation. Consequently, the MM@TMV system successfully coupled metabolic support with robust effector licensing and enabled complete tumor regression when combined with TCR-T cells in refractory models.

As with any bacteria-enhanced immunotherapy, systemic safety remains a major concern for clinical translation. In our study, the engineered bacteria preferentially colonized hypoxic tumors and were gradually cleared from major organs, including the heart, liver, spleen, lungs, and kidneys. Comprehensive physiological monitoring showed no significant body weight loss, hematological abnormalities, or hepatic toxicity. These results support favorable short-term tolerability in mice. However, these findings do not fully exclude clinically relevant risks in humans. CAR-T therapy itself carries a risk of cytokine release syndrome. Therefore, its combination with systemically administered Gram-negative bacteria may further amplify inflammatory cytokine release or induce bacteremia, endotoxin-driven systemic inflammation, or sepsis, despite membrane coating. Accordingly, additional preclinical studies are required before clinical translation. These studies should define cytokine kinetics, bacterial persistence, endotoxin-related toxicity, repeat-dose tolerance, and dose-escalation limits for combination strategy.

Ultimately, the validation of MM@TMV in a humanized PDX model of Claudin 18.2-positive GC highlights its broad translational potential. Programmable engineered bacteria can spatiotemporally couple metabolic rewiring with enhanced antigen presentation. This strategy provides a highly modular adjuvant platform for unlocking the curative potential of cellular immunotherapies in solid tumors.

## Materials and methods

### Ethics statements

All animal care and experimental protocols were approved by the Institutional Animal Care and Use Committee (IACUC) of Sun Yat-sen University (No. SYSU-IACUC-2023-B0965) by the standards of the National Institutes of Health (SYSU-IACUC-2025-000838). All mice were housed under controlled environmental conditions with 21°C–25°C, 40%–60% relative humidity, and a 12-h/12-h light/dark cycle. The study complied with the ethics committee’s limit on maximal tumor size (1,500 mm^3^) and the requirement that mouse body weight loss did not exceed 20%. Neither of these thresholds was exceeded at any point during the study. The use of human blood samples was approved by the Research Ethics Committee of The Seventh Affiliated Hospital of Sun Yat-sen University (KY-2025-348-01).

### Mice and cell lines

OT-1 mice were obtained from Jackson Laboratory (Bar Harbor, USA) and bred in-house. NCG (NOD/ShiLtJGpt-*Prkdc*^*em26Cd52*^*Il2rg*^*em26Cd22*^/Gpt) male and C57BL/6 and BALB/c female mice (6 weeks old) were purchased from GemPharmatech (Jiangsu, P.R. China). Mice were housed in a specific pathogen-free room with sufficient feed and drinking water. All animal experimental protocols were reviewed and approved by the Institutional Animal Care and Use Committee, Sun Yat-sen University (Approval numbers SYSU-IACUC-2025-000838). Animal experiments were performed in accordance with the NIH Guide for the Care and Use of Laboratory Animals (NIH Publication No. 85–23, Rev. 1985). B16F10, 4T1, Panc-02, MKN45, and HGC cell lines were purchased from the American Type Culture Collection (ATCC). YTN-16 was a gift given by Pro. Sachiyo Nomura from University of Tokyo.^50^ B16-OVA-luciferase and Panc-02-OVA-luciferase (B16F10-OVA-luc and Panc-02-OVA-luc, B16F10 and Panc-02 transfected by lentivirus to express OVA and luciferase, followed by puromycin selection), as well as HGC-Claudin18.2 (HGC cells transduced with lentivirus to express Claudin18.2 and selected with puromycin), were cultured in complete DMEM medium (Yeasen, 41401ES76) supplemented with 10% fetal bovine serum (FBS, Yeasen, 40131ES76) and 100 U/mL penicillin-streptomycin (P/S, Corning, 30-002-CI).

### Murine T cell culture and activation

Spleens harvested from OT-1 mice were cut and ground into a single-cell suspension in cold PBS, which were lysed with ACK lysis buffer (Yeasen, 40401ES60). The obtained splenocytes were resuspended in a T cell culture medium (TCM). TCM consisted of complete RPMI1640 medium (Yeasen, 41402ES76), 10% FBS, 100 U/mL penicillin, 0.1 mg/mL streptomycin, 10 mM HEPES, and 1 mM sodium pyruvate. Murine IL-2 (10 ng/mL), murine IL-7 (1 ng/mL), and OVA_257-264_ peptide (1 μg/mL) were also supplemented for T cell activation. After culturing for 2 days at 37°C, live cells were enriched via density gradient centrifugation in Ficoll-Paque Plus (230 × *g* for 15 min with brake off) and cultured for another 2 days in TCM containing IL-2 and IL-7 (10 ng/mL).

### CAR-T cell production protocol

For optimal lentivirus production, HEK293T cells were transfected with CAR vectors, psPAX2 (Addgene#12260), and pMD2.G (Addgene#12259) packaging plasmids using Hieff Trans Liposomal Transfection Reagent (Yeasen) (Table S1). Virus-containing supernatant was harvested 48 and 72 h post transfection, centrifuged (1000 × *g*) for 5 min, and filtered through 0.45-μm cellulose acetate membrane. The virus solution was then concentrated by ultracentrifugation (25,000 × *g*) at 4°C for 2 h and resuspended in a serum-free medium. Peripheral blood mononuclear cells (PBMCs) were obtained from healthy adult donors. PBMCs were isolated by density gradient centrifugation using Lymphocyte Separation Medium (Corning) and subsequently activated with ImmunoCult Human CD3/CD28 T cell Activator (STEMCELL) in TCM (OptiVitro T cell Medium SF, ExCell Bio) supplemented with 200 U/mL recombinant human IL-2. After 48 h, T cells were collected and transduced with lentiviral supernatant (MOI = 20) with 8 mg/mL polybrene (Yeasen, 40804ES76). After 24 h, the viral supernatant was replaced with fresh CTL medium containing 200 U/mL human IL-2.

### Preparation of hypoxia-responsive engineered bacteria

To generate D-mannose isomerase-expressing *E. coli MG1655*, the gene encoding D-mannose isomerase, along with the pPepT tandem promoter and the N-terminal pelB secretion signal sequence, was inserted into a pET28a plasmid to construct the hypoxia-sensitive plasmid pET28a-D-mannose isomerase. Additionally, a hypoxia-sensitive plasmid expressing *mCherry* (pET28a-mCherry) was constructed to facilitate the detection of gene expression. Next, both pET28a-D-mannose isomerase and pET28a-mCherry plasmids were chemically transformed into the *E. coli MG1655* strain to obtain the hypoxia-responsive engineered strain MG-MIase. All engineered bacteria were cultured in Luria-Bertani broth medium at 37°C (Table S2).

### Hypoxia-responsive performance of MG-MIase

The expression level of D-mannose isomerase protein was evaluated using WB and ELISA. The *MG1655* and MG-MIase strains were divided into four groups to determine whether MG-MIase possesses hypoxia-responsive capabilities: MG under normoxic conditions, MG-MIase under normoxic conditions, MG under hypoxic conditions, and MG-MIase under hypoxic conditions. To further assess the response of the engineered bacteria to varying degrees of hypoxia, each group was exposed to different oxygen concentrations: 21% (normoxia), 12%, 6%, and 0%. All groups were adjusted to a bacterial concentration of 5 × 10^8^ CFU. After 12 h of incubation under the respective conditions, corresponding characterizations were performed. Additionally, engineered bacteria carrying the pET28a-mCherry plasmid were used in bioluminescence imaging experiments to evaluate the accuracy of their hypoxia response.

### MM@TMV construction and characterization

Briefly, tumor cells were incubated with DSPE-PEG2000-NHS (final concentration: 1 μg/mL) at 37°C for 30 min. Subsequently, human- and mouse-derived tumor cell membranes were extracted using a Membrane and Cytoplasmic Protein Extraction Kit (Beyotime, P0033). TMVs were then prepared by repeatedly extruding the membranes 20 times through a 200-nm polycarbonate membrane (using an LF-1 lipid extruder, Avestin, equipped with a 400-nm membrane). Next, 100 μL of TMVs were mixed with 1 mL of *E. coli* MG-MIase solution (10^8^ CFU/mL) and gently stirred at 4°C for 18 h. After centrifuging (× 8,000 rpm) three times, the precipitate was resuspended in 1 mL of PBS to obtain MM@TMV. The morphology of MG-MIase and MM@TMV was observed using TEM and SEM.

### Bacterial tumor-specific colonization and biodistribution *in vivo*

To evaluate the tumor-targeting ability of the engineered bacteria, MG-*mCherry* was administered to B16F10 tumor-bearing mice via either *i.v.* or *i.t.* injection. At designated time points (days 0, 1, 2, and 3), tumors and other organs were harvested for *ex vivo* bioluminescence imaging using the IVIS optical imaging system. For bacterial colony counting, subcutaneous B16F10 tumor-bearing mice with tumor volumes of approximately 100 mm^3^ were randomly divided into two groups (*n* = 5 per group) and injected with MG-MIase via either *i.v.* or *i.t.* routes. At specific time points (days 0, 1, 3, and 7), the tumor, heart, liver, spleen, lung, and kidneys were collected and homogenized under sterile conditions using a tissue dissociator. The resulting homogenates were serially diluted with PBS and plated on LB agar plates. Colonies were counted after incubation at 37°C for 24 h.

### Evaluation of the D-mannose production capability catalyzed by MG-MIase

MG-MIase (1 × 10^6^ CFU) was added to culture medium containing D-fructose (10 g/L) and incubated at 37°C. At designated time points (0, 2, 4, 6, and 8 h), the culture supernatant was collected to measure the concentration of D-mannose.

### Evaluation of *in vivo* D-mannose production capability catalyzed by MG-MIase

Subcutaneous 4T1 tumor-bearing mice with tumor volumes of approximately 100 mm^3^ were randomly assigned to two groups (*n* = 5 per group) and subcutaneously injected with 5 × 10^6^ CFU MG or MG-MIase. 1 day later, tumors were collected and homogenized, and the intratumoral D-mannose content was measured.

### Evaluation of MG-MIase-mediated tumor growth inhibition

B16F10, MKN45, 4T1, and Panc-02 tumor cell lines were seeded into 96-well plates at a density of 1 × 10^4^ cells per well. After 12 h of incubation, the medium was replaced with tumor cell culture medium containing 100 nM D-fructose, followed by the addition of 1 × 10^4^ CFU of MG or MG-MIase to each well for continued incubation. In the D-mannose group, 25 nM D-mannose was directly added to the culture medium. Cell viability was assessed using the MTT assay at 0, 24, 48, 72, and 96 h.

### Evaluation of T cell stemness and exhaustion phenotypes

T cells were resuspended in culture medium containing 100 nM D-fructose and seeded into 96-well plates at a density of 1 × 10^4^ cells per well. Subsequently, 1 × 10^4^ CFU of MG or MG-MIase were added to each well for continued incubation. For the D-mannose group, 20 nM D-mannose was added to the T cells for further culture. After 3 days, flow cytometry was performed to evaluate changes in T cell stemness and exhaustion phenotypes.

### RNA-seq and analysis

T cells were resuspended in culture medium containing 100 nM D-fructose and seeded into 96-well plates at a density of 1 × 10^4^ cells per well. Subsequently, 1 × 10^4^ CFU of MG or MG-MIase was added to each well for continued incubation. For the D-mannose group, 20 nM D-mannose was added to the T cells for further culture. After 3 days, T cells were collected, and total RNA was extracted using Trizol (Invitrogen, Carlsbad). Samples were sent to Lc-bio Co. for RNA sequencing following the standard protocol, and data were analyzed via the online platform (https://www.omicstudio.cn/home).

### *In vivo* biosafety evaluation of MG-MIase

Subcutaneous B16F10 tumor-bearing mice with tumor volumes of approximately 100 mm^3^ were randomly assigned to two groups (*n* = 5 per group) and injected with MG-MIase either intravenously or intratumorally. At specified time points (days 0, 1, 3, and 7), the tumor and major organs (heart, liver, spleen, lung, and kidney) were harvested for pathological analysis.

### Seahorse metabolic assay

Tumor-infiltrating TCR-T cells from the B16F10-OVA tumor model were first enriched by Percoll (GE Healthcare) density-gradient centrifugation and stained with surface antibodies and DAPI (Sigma-Aldrich). The cells were then sorted using an Aria II cell sorter (BD Biosciences) for subsequent Seahorse metabolic analysis. Seahorse assays were performed to measure the OCR and ECAR of CAR-T cells. TCR-T cells subjected to different treatment conditions (3 × 10^5^ cells per well) were seeded into a Seahorse culture plate (Seahorse Bioscience) and incubated at 37°C in a non-CO_2_ incubator for 40 min. OCR and ECAR were then measured using a Seahorse XFe96 Analyzer (Seahorse Bioscience) according to the manufacturer’s instructions. During the assay, cells were sequentially treated with oligomycin (1 μM; Sigma-Aldrich), carbonyl cyanide-4-(trifluoromethoxy) phenylhydrazone (2 μM; Sigma-Aldrich), rotenone (0.5 μM; Sigma-Aldrich), antimycin A (0.5 μM; Sigma-Aldrich), glucose (10 mM; Sigma-Aldrich), and 2-deoxy-D-glucose (50 mM; Sigma-Aldrich). Three replicates were included for each condition within a single experiment.

### Flow cytometry analysis

Cells were first stained with LIVE/DEAD Fixable Aqua dye to assess viability, followed by washing with PBS containing 0.2% (w/v) bovine serum albumin. Fc receptors were then blocked, and cells were incubated with the indicated surface antibodies at 4°C for 30 min. After incubation, cells were washed again and resuspended in the same buffer for flow cytometry analysis. For intracellular cytokine staining, cells were stimulated at 37°C for 4 h with a cell stimulation cocktail containing protein transport inhibitors (Invitrogen/Thermo Fisher Scientific). After stimulation, live/dead staining and surface staining procedures were followed. Cells were then fixed and permeabilized using the Foxp3/Transcription Factor Staining Buffer Set (eBioscience) according to the manufacturer’s instructions, followed by incubation with the indicated intracellular antibodies at room temperature for 30 min. After staining, cells were washed and resuspended in buffer for flow cytometric analysis.

The following antibodies used in flow cytometry (FCM) were purchased from BioLegend: anti-mouse PD-L1 (APC, 124312), anti-human TCF1 (PE, 655208), anti-mouse LAG-3 (APC, 125210), anti-mouse PD-1 (APC/Cy7, 135224), anti-mouse Tim-3(PE/Cy7, 119715), anti-human CD45RO (Percp, 304251), anti-human Tim-3(APC, 364804), anti-human PD-1 (BV421, 329919), anti-human TNF-α (FITC, 502906), anti-human IFN-γ (PE/Cy7, 506517), anti-mouse CD4 (APC/Cy7, 100414), anti-mouse CD8a (FITC, 300906; PE, 162304; APC/Cy7, 100714; PE/Cy7 100722), anti-mouse granzyme B (FITC, 515403), anti-human CD8 (APC/Cy7, 344713; PE/Cy7, 344750), anti-mouse CD11c (PE/Cy7, 117318; PerCP/Cy5.5, 117328), anti-mouse CD44 (PE, 103008), anti-mouse CD45 (PE, 103106; APC/Cy7, 103116; FITC, 103108), anti-human CD45 (PE, 982322; PerCP, 982318), anti-mouse CD80 (APC, 104714; PE-Cy7, 104734), anti-mouse CD62L (FITC, 104406; APC/Cy7, 104428), anti-mouse CD86 (FITC, 105006; PE, 105008; APC/Cy7, 105030), anti-mouse CD206 (APC, 141708), anti-mouse F4/80 (FITC, 123108; PE/Cy7, 123114), anti-mouse TCR Vα2 (PE, 127807), anti-mouse H-2K^b^ SIINFEKL (PE, 141603), anti-mouse NK1.1 (APC/Cy7, 108724), anti-mouse PD-L1 (APC, 124312), anti-human PD-L1 (APC, 393610), and anti-mouse IFN-γ (PE, 505808).

The following antibodies used in flow cytometry were purchased from BD Biosciences: anti-mouse TCF1 (BV421, 566692).

For proliferation studies, CD8^+^ TILs and CAR-T cells were stained with CFSE before *in vitro* co-culture experiments with tumor cells or *in vivo* T cell proliferation assays.

### Assessment of T cell cytotoxicity

1 × 10^4^ tumor cells were seeded into flat-bottom 96-well tissue culture plates and incubated overnight at 37°C. The next day, 0.25 × 10^4^ to 2 × 10^4^ CAR-T or TCR-T cells (effector-to-target ratio [E:T] = 0.25:1 to 2:1) were added to each well, along with PBS, MG-MIase (normoxia), or MG-MIase (hypoxia), and cultured in medium containing 100 nM fructose for 24 h. To detect lysed tumor cells, 100 μL of Bright-Lite luciferase substrate (Vazyme, DD1209-02) was added to each well. After a 5-min incubation, luminescent signals were measured using a microplate reader (Tecan). The degree of specific lysis of target cancer cells was determined by comparing the proportion of surviving target cells to that in the control group.

### Evaluation of the phagocytosis of MM@TMV by BMDCs

TMVs and engineered bacteria were labeled with Cy7 and FITC, respectively, after which TMV was conjugated onto the engineered bacteria. Then, 1 × 10^4^ CFUs of MM@TMV were co-incubated with 1 × 10^4^ BMDCs for 24 h. After incubation, the cells were collected and analyzed by flow cytometry to evaluate the phagocytosis of MM@TMV by DCs. In another parallel experiment, D-fructose (25 nM) was added to the culture medium. Supernatants were collected at different time points to measure the D-mannose content, thereby evaluating the activity of the engineered bacteria.

### Animal experiments

For the immunodeficient mouse model, 1 million B16F10 or Panc-02 cells suspended in 100 μL sterile PBS were subcutaneously injected into the flank of C57BL/6 mice, and 1 million 4T1 cells in 100 μL PBS were injected into the flank of BALB/c mice. When tumors reached 60–70 mm^3^, the mice were randomly divided into two groups. 1 day prior to treatment, mice received 10 Gy total body irradiation to deplete lymphocytes. 5 million MG or MG-MIase were suspended in 20 μL sterile PBS and administered via i.t. injection. Tumor volume and body weight were measured every 2 days. The experiment was terminated when tumors reached 1,500 mm^3^. Mice were euthanized, and tumors were collected and weighed.

For the immunocompetent mouse model experiments, 1 million B16F10 or Panc-02 cells in 100 μL PBS were injected subcutaneously into the flank of C57BL/6 mice and 1 million 4T1 cells into BALB/c mice. When tumors reached to 60–70 mm^3^, mice were treated intratumorally with PBS, 5 million MG, or 5 million MG-MIase (20 μL in sterile PBS). Tumor volume and body weight were measured every 2 days, and survival was monitored.

For the *i.t.* injection of MM@TMV experiments, when tumors reached 60–70 mm^3^, mice were randomly divided into four groups and treated intratumorally with PBS, MG-MIase, MG@TMV, or MM@TMV (20 μL in sterile PBS). Tumor volume and body weight were monitored every 2 days, and survival was monitored.

For the combination therapy of MM@TMV with TCR-T cells experiments, 1 million B16-OVA or Panc-02-OVA cells suspended in 100 μL PBS were injected subcutaneously into the flank of C57BL/6 mice. When tumors reached 60–70 mm^3^, mice were randomly divided into five groups: G1: PBS; G2: TCR-T cells; G3: TCR-T cells + MG-MIase; G4: TCR-T cells + MG@TMV; G5: TCR-T cells + MM@TMV. 1 day before treatment, mice received intraperitoneal injection of cyclophosphamide (2 mg). G1 mice received 100 μL PBS via tail vein, while G2–G5 mice received 5 × 10^6^ OT-I CD8^+^ T cells intravenously, followed by *i.t.* injection of PBS, 5 million MG-MIase, 5 million MG@TMV, or 5 million MM@TMV, respectively. Tumor volume and body weight were measured every 2 days, and survival was recorded.

For the lung metastasis model using MM@TMV in combination with TCR-T cells, 0.5 million Panc-OVA-luc cells were intravenously injected into C57BL/6 mice (100 μL PBS). On day 6, mice received intraperitoneal cyclophosphamide (2 mg), followed by treatment the next day as described above. 10 min after intraperitoneal injection of 100 μL D-Luciferin, Potassium Salt D (Yeasen, 40902ES03) at a concentration of 30 mg/mL, tumor growth was monitored by bioluminescence imaging on an IVIS spectrum imaging system, and survival of mice was recorded. In another parallel experiment, mice were euthanized at 8 days, and lung tissues were harvested for assessment of tumor nodules.

To establish the gastric orthotopic tumor model, mice were anesthetized with isoflurane and shaved around the abdomen. An incision was made to expose the stomach. YTN16-OVA cells (5 × 10^6^) in 30 μL Matrigel (25% in PBS) were injected into the serosa of the stomach, followed by suturing of the incision. Mice were injected with MM@TMV and/or TCR-T cells starting from the 4th week after surgery and sacrificed on the 8th week.

For the experiment combining MM@TMV with CAR-T cells, mice were first irradiated, followed by tail vein injection of HSCs the next day. At week 7 post transplantation, mice with more than 60% huCD45^+^ cells in peripheral blood were selected for subsequent studies. By week 12, immune cell differentiation was complete. For tumor implantation, one million HGC-Claudin18.2-luc cells (Table S2) suspended in 100 μL sterile PBS were subcutaneously injected into one flank of NCG (NOD/ShiLtJGpt-*Prkdc*^*em26Cd52*^*Il2rg*^*em26Cd22*^/Gpt) mice. When tumor volumes reached 60–70 mm^3^, the mice were randomly divided into five groups: G1: PBS; G2: CAR-T cells; G3: CAR-T cells with MG-MIase; G4: CAR-T cells with MG@TMV; G5: CAR-T cells with MM@TMV. 1 day prior to treatment, all mice received intraperitoneal injection of cyclophosphamide (2 mg). G1 mice received 100 μL PBS via tail vein, while mice in G2–G5 groups received 5 × 10^6^ CAR-T cells intravenously, followed by i.t. injection of PBS, 5 million MG-MIase, 5 million MG@TMV, or 5 million MM@TMV, respectively. Bioluminescence imaging was performed every 2 days to monitor tumor signals, and mouse survival was recorded. For another cohort bearing human-derived tumor tissues, the procedure was the same, with tumor volume measured every 2 days, and survival of mice was recorded.

### ELISPOT assay

Tumor cells were irradiated with 120 Gy and then digested into a single-cell suspension. Tumor tissues from mice were also dissociated into single-cell suspensions, and tumor-infiltrating CAR-T cells were sorted by flow cytometry. A total of 4 × 10^5^ CAR-T cells were mixed with 2.5 × 10^4^ irradiated tumor cells in 200 μL of complete medium and seeded into a 96-well ELISPOT plate pre-coated with IFN-γ capture antibodies (BD IFN-γ ELISPOT kit). The plate was wrapped in foil and incubated at 37°C for 24 h. After incubation, the plates were developed according to the manufacturer’s protocol. Plates were scanned using a CTL ImmunoSpot plate reader, and the data were analyzed with CTL ImmunoSpot software.

### Statistical analysis

For quantitative analysis, at least three biological replicates were performed. All statistical analyses were conducted using GraphPad Prism 10 software, and data are presented as mean ± standard deviation (mean ± SD). Comparisons between two groups were performed using unpaired *t* tests, while multiple group comparisons were analyzed by ordinary one-way ANOVA. Tumor growth curves were analyzed using two-way ANOVA. Differences in survival curves were evaluated by the log rank test.

## Data and code availability

The data that support the findings of this study are available from the corresponding author upon reasonable request.

## Acknowledgments

This work was financially supported by Shenzhen Medical Research Fund (A2403016), Guangdong Basic and Applied Basic Research (2026A1515011271), Shenzhen Science and Technology (no. JCYJ20240813150317023), Research Start-up Fund of Post-doctoral of SAHSYSU (no. ZSQYRSFPD0086), 10.13039/501100012151Sanming Project of Medicine in Shenzhen (no. SZSM202411013), Shenzhen Medical Research Fund (A2403055), Shenzhen Science and Technology Program (JCYJ20240813150412017), and Postgraduate Research & Practice Innovation Program of Jiangsu Province (KYCX24_3349).

## Author contributions

Conceptualization: A.D., C.Z., S.Y., Z.Z. Methodology: J.C., X.L. Investigation: T.L., H.Z. Visualization: J.C., X.L., A.D. Supervision: A.D., C.Z., S.Y., Z.Z. Writing – original draft: J.C., A.D. Writing – review and editing: C.Z., A.D. YTN-16 cell line: S.N.

## Declaration of interests

The authors declare no competing interests.

## Declaration of generative AI and AI-assisted technologies in the writing process

During the preparation of this work, we used ChatGPT (Open AI, GPT-5.1) to improve language fluency, readability, and overall expression. After using this tool, we thoroughly reviewed and edited the content as needed and take full responsibility for the content of this publication.

## Supplemental information

Document S1. Figures S1–S14 and Tables S1 and S2

Document S2. Article plus supplemental information

## References

[1] June C.H., O'Connor R.S., Kawalekar O.U., Ghassemi S., Milone M.C. (2018). CAR T cell immunotherapy for human cancer. Science.

[2] Milone M.C., Xu J., Chen S.J., Collins M.A., Zhou J., Powell D.J., Melenhorst J.J. (2021). Engineering enhanced CAR T-cells for improved cancer therapy. Nat. Cancer.

[3] Finck A.V., Blanchard T., Roselle C.P., Golinelli G., June C.H. (2022). Engineered cellular immunotherapies in cancer and beyond. Nat. Med..

[4] Uslu U., June C.H. (2025). Beyond the blood: expanding CAR T cell therapy to solid tumors. Nat. Biotechnol..

[5] Albelda S.M. (2024). CAR T cell therapy for patients with solid tumours: key lessons to learn and unlearn. Nat. Rev. Clin. Oncol..

[6] Shah N.N., Fry T.J. (2019). Mechanisms of resistance to CAR T cell therapy. Nat. Rev. Clin. Oncol..

[7] Rafiq S., Hackett C.S., Brentjens R.J. (2020). Engineering strategies to overcome the current roadblocks in CAR T cell therapy. Nat. Rev. Clin. Oncol..

[8] Zebley C.C., Zehn D., Gottschalk S., Chi H. (2024). T cell dysfunction and therapeutic intervention in cancer. Nat. Immunol..

[9] Dimitri A.J., Baxter A.E., Chen G.M., Hopkins C.R., Rouin G.T., Huang H., Kong W., Holliday C.H., Wiebking V., Bartoszek R. (2024). TET2 regulates early and late transitions in exhausted CD8(+) T cell differentiation and limits CAR T cell function. Sci. Adv..

[10] Pishesha N., Harmand T.J., Ploegh H.L. (2022). A guide to antigen processing and presentation. Nat. Rev. Immunol..

[11] Tang L., Pan S., Wei X., Xu X., Wei Q. (2023). Arming CAR-T cells with cytokines and more: Innovations in the fourth-generation CAR-T development. Mol. Ther..

[12] Du B., Qin J., Lin B., Zhang J., Li D., Liu M. (2025). CAR-T therapy in solid tumors. Cancer Cell.

[13] Zhao Z., Li Q., Qu C., Jiang Z., Jia G., Lan G., Luan Y. (2025). A collagenase nanogel backpack improves CAR-T cell therapy outcomes in pancreatic cancer. Nat. Nanotechnol.

[14] Zhong W., Qin Z., Yu Z., Yang J., Yan D., Engel N.W., Sheppard N.C., Fan Y., Radhakrishnan R., Xu X. (2025). Overcoming extracellular vesicle-mediated fratricide improves CAR T cell treatment against solid tumors. Nat. Cancer.

[15] Fernandez M.R., Cleveland J.L. (2018). Metabolic Reprogramming via Targeting CD38 NADase Augments Adoptive T Cell Therapy. Cell Metab..

[16] Guerrero J.A., Klysz D.D., Chen Y., Malipatlolla M., Lone J., Fowler C., Stuani L., May A., Bashti M., Xu P. (2024). GLUT1 overexpression in CAR-T cells induces metabolic reprogramming and enhances potency. Nat. Commun..

[17] Jin H., Wu P., Lv C., Zhang S., Zhang Y., Li C., Gao R., Shan G., Bi H., Chang H. (2025). Mannose inhibits PKM2 lactylation to induce pyroptosis in bladder cancer and activate antitumor immune responses. Commun. Biol..

[18] Chaube B., Bhat M.K. (2016). AMPK, a key regulator of metabolic/energy homeostasis and mitochondrial biogenesis in cancer cells. Cell Death Dis..

[19] Zhang C.S., Hawley S.A., Zong Y., Li M., Wang Z., Gray A., Ma T., Cui J., Feng J.W., Zhu M. (2017). Fructose-1,6-bisphosphate and aldolase mediate glucose sensing by AMPK. Nature.

[20] Gonzalez P.S., O'Prey J., Cardaci S., Barthet V.J.A., Sakamaki J.I., Beaumatin F., Roseweir A., Gay D.M., Mackay G., Malviya G. (2018). Mannose impairs tumour growth and enhances chemotherapy. Nature.

[21] Sha J., Cao D., Cui R., Xia L., Hua X., Lu Y., Han S. (2020). Mannose Impairs Lung Adenocarcinoma Growth and Enhances the Sensitivity of A549 Cells to Carboplatin. Cancer Manag. Res..

[22] Harada Y., Mizote Y., Suzuki T., Hirayama A., Ikeda S., Nishida M., Hiratsuka T., Ueda A., Imagawa Y., Maeda K. (2023). Metabolic clogging of mannose triggers dNTP loss and genomic instability in human cancer cells. Elife.

[23] Qiu Y., Su Y., Xie E., Cheng H., Du J., Xu Y., Pan X., Wang Z., Chen D.G., Zhu H. (2025). Mannose metabolism reshapes T cell differentiation to enhance anti-tumor immunity. Cancer Cell.

[24] Greco B., Malacarne V., De Girardi F., Scotti G.M., Manfredi F., Angelino E., Sirini C., Camisa B., Falcone L., Moresco M.A. (2022). Disrupting N-glycan expression on tumor cells boosts chimeric antigen receptor T cell efficacy against solid malignancies. Sci. Transl. Med..

[25] Marriott B.P., Cole N., Lee E. (2009). National estimates of dietary fructose intake increased from 1977 to 2004 in the United States. J. Nutr..

[26] Schild T., Wallisch P., Zhao Y., Wang Y.T., Haughton L., Chirayil R., Pierpont K., Chen K., Nunes-Violante S., Cross J. (2025). Metabolic engineering to facilitate anti-tumor immunity. Cancer Cell.

[27] Hu X., Zhang P., Miao M., Zhang T., Jiang B. (2016). Development of a recombinant d-mannose isomerase and its characterizations for d-mannose synthesis. Int. J. Biol. Macromol..

[28] Gurbatri C.R., Arpaia N., Danino T. (2022). Engineering bacteria as interactive cancer therapies. Science.

[29] Redenti A., Im J., Redenti B., Li F., Rouanne M., Sheng Z., Sun W., Gurbatri C.R., Huang S., Komaranchath M. (2024). Probiotic neoantigen delivery vectors for precision cancer immunotherapy. Nature.

[30] Yang S., Sheffer M., Kaplan I.E., Wang Z., Tarannum M., Dinh K., Abdulhamid Y., Bobilev E., Shapiro R., Porter R. (2024). Non-pathogenic E. coli displaying decoy-resistant IL18 mutein boosts anti-tumor and CAR NK cell responses. Nat. Biotechnol..

[31] Wu Y., Liu B., Yan Y., Gong C., Wang K., Liu N., Zhu Y., Li M., Wang C., Yang Y. (2024). Thermal-responsive activation of engineered bacteria to trigger antitumor immunity post microwave ablation therapy. Nat. Commun..

[32] Qiao C., Wang L., Huang C., Jia Q., Bao W., Guo P., Tan D., Chen Z., Shi C., Rao Z. (2025). Engineered Bacteria Manipulate Cysteine Metabolism to Boost Ferroptosis-Based Pancreatic Ductal Adenocarcinoma Therapy. Adv. Mater..

[33] Xie X.T., Guan M., Cheng K., Li Y., Zhang B., Zhou Y.T., Tan L.F., Dong P.S., Chen S., Liu B. (2025). Programmable engineered bacteria as sustained-releasing antibody factory in situ for enhancing tumor immune checkpoint therapy. Sci. Adv..

[34] Adu-Berchie K., Brockman J.M., Liu Y., To T.W., Zhang D.K.Y., Najibi A.J., Binenbaum Y., Stafford A., Dimitrakakis N., Sobral M.C. (2023). Adoptive T cell transfer and host antigen-presenting cell recruitment with cryogel scaffolds promotes long-term protection against solid tumors. Nat. Commun..

[35] Wauters A.C., Scheerstra J.F., Vermeijlen I.G., Hammink R., Schluck M., Woythe L., Wu H., Albertazzi L., Figdor C.G., Tel J. (2022). Artificial Antigen-Presenting Cell Topology Dictates T Cell Activation. ACS Nano.

[36] Xu J., Cao W., Wang P., Liu H. (2022). Tumor-Derived Membrane Vesicles: A Promising Tool for Personalized Immunotherapy. Pharmaceuticals (Basel).

[37] Bommireddy R., Munoz L.E., Kumari A., Huang L., Fan Y., Monterroza L., Pack C.D., Ramachandiran S., Reddy S.J.C., Kim J. (2020). Tumor Membrane Vesicle Vaccine Augments the Efficacy of Anti-PD1 Antibody in Immune Checkpoint Inhibitor-Resistant Squamous Cell Carcinoma Models of Head and Neck Cancer. Vaccines (Basel).

[38] Busuttil R.A., Liu D.S., Di Costanzo N., Schröder J., Mitchell C., Boussioutas A. (2018). An orthotopic mouse model of gastric cancer invasion and metastasis. Sci. Rep..

[39] Qi C., Liu C., Gong J., Liu D., Wang X., Zhang P., Qin Y., Ge S., Zhang M., Peng Z. (2024). Claudin18.2-specific CAR T cells in gastrointestinal cancers: phase 1 trial final results. Nat. Med..

[40] Tao D., Guan B., Li Z., Jiao M., Zhou C., Li H. (2023). Correlation of Claudin18.2 expression with clinicopathological characteristics and prognosis in gastric cancer. Pathol. Res. Pract..

[41] McPhedran S.J., Carleton G.A., Lum J.J. (2024). Metabolic engineering for optimized CAR-T cell therapy. Nat. Metab..

[42] Reinhard K., Rengstl B., Oehm P., Michel K., Billmeier A., Hayduk N., Klein O., Kuna K., Ouchan Y., Wöll S. (2020). An RNA vaccine drives expansion and efficacy of claudin-CAR-T cells against solid tumors. Science.

[43] Niu X., Zhang P., Dai L., Peng X., Liu Z., Tang Y., Zhang G., Wan X. (2025). Flagellin engineering enhances CAR-T cell function by reshaping tumor microenvironment in solid tumors. J. Immunother. Cancer.

[44] Del Prete A., Salvi V., Soriani A., Laffranchi M., Sozio F., Bosisio D., Sozzani S. (2023). Dendritic cell subsets in cancer immunity and tumor antigen sensing. Cell. Mol. Immunol..

[45] Liang C., Yuan Z., Yang S., Zhu Y., Chen Z., Can D., Lei A., Li H., Leng L., Zhang J. (2025). Mannose Promotes beta-Amyloid Pathology by Regulating BACE1 Glycosylation in Alzheimer's Disease. Adv. Sci..

[46] Xiao P., Hu Z., Lang J., Pan T., Mertens R.T., Zhang H., Guo K., Shen M., Cheng H., Zhang X. (2023). Mannose metabolism normalizes gut homeostasis by blocking the TNF-alpha-mediated proinflammatory circuit. Cell. Mol. Immunol..

[47] Fowle-Grider R., Rowles J.L., Shen I., Wang Y., Schwaiger-Haber M., Dunham A.J., Jayachandran K., Inkman M., Zahner M., Naser F.J. (2024). Dietary fructose enhances tumour growth indirectly via interorgan lipid transfer. Nature.

[48] Fang J.H., Chen J.Y., Zheng J.L., Zeng H.X., Chen J.G., Wu C.H., Cai J.L., Wang Z.Y., Zhuang S.M. (2023). Fructose Metabolism in Tumor Endothelial Cells Promotes Angiogenesis by Activating AMPK Signaling and Mitochondrial Respiration. Cancer Res..

[49] Yan H., Wang Z., Teng D., Chen X., Zhu Z., Chen H., Wang W., Wei Z., Wu Z., Chai Q. (2024). Hexokinase 2 senses fructose in tumor-associated macrophages to promote colorectal cancer growth. Cell Metab..

[50] Yamamoto M., Nomura S., Hosoi A., Nagaoka K., Iino T., Yasuda T., Saito T., Matsushita H., Uchida E., Seto Y. (2018). Established gastric cancer cell lines transplantable into C57BL/6 mice show fibroblast growth factor receptor 4 promotion of tumor growth. Cancer Sci..

