# Supplementary material for: Programmable engineered bacteria manipulate metabolism and remodel the TME in situ for enhancing adoptive cell therapy
Source: Mol Ther. 2026 Jul 9;34(9):5222–42. doi: 10.1016/j.ymthe.2026.06.042 (PMC13555542; doi:10.1016/j.ymthe.2026.06.042)
Supplement: Supplementary file 1 [file mmc1.pdf]

YMTHE, Volume *34*

Supplemental Information

Programmable engineered bacteria manipulate metabolism and remodel the TME *in situ* for enhancing adoptive cell therapy

Jin Chen, Tianliang Liu, Xiumin Liu, Hongyue Zhang, Chen Wang, Yuan Meng, Miaoqing Wu, Sachiyo Nomura, Zhe Zhang, Songcheng Yin, Changhua Zhang, and Aoran Dong

Supplemental File

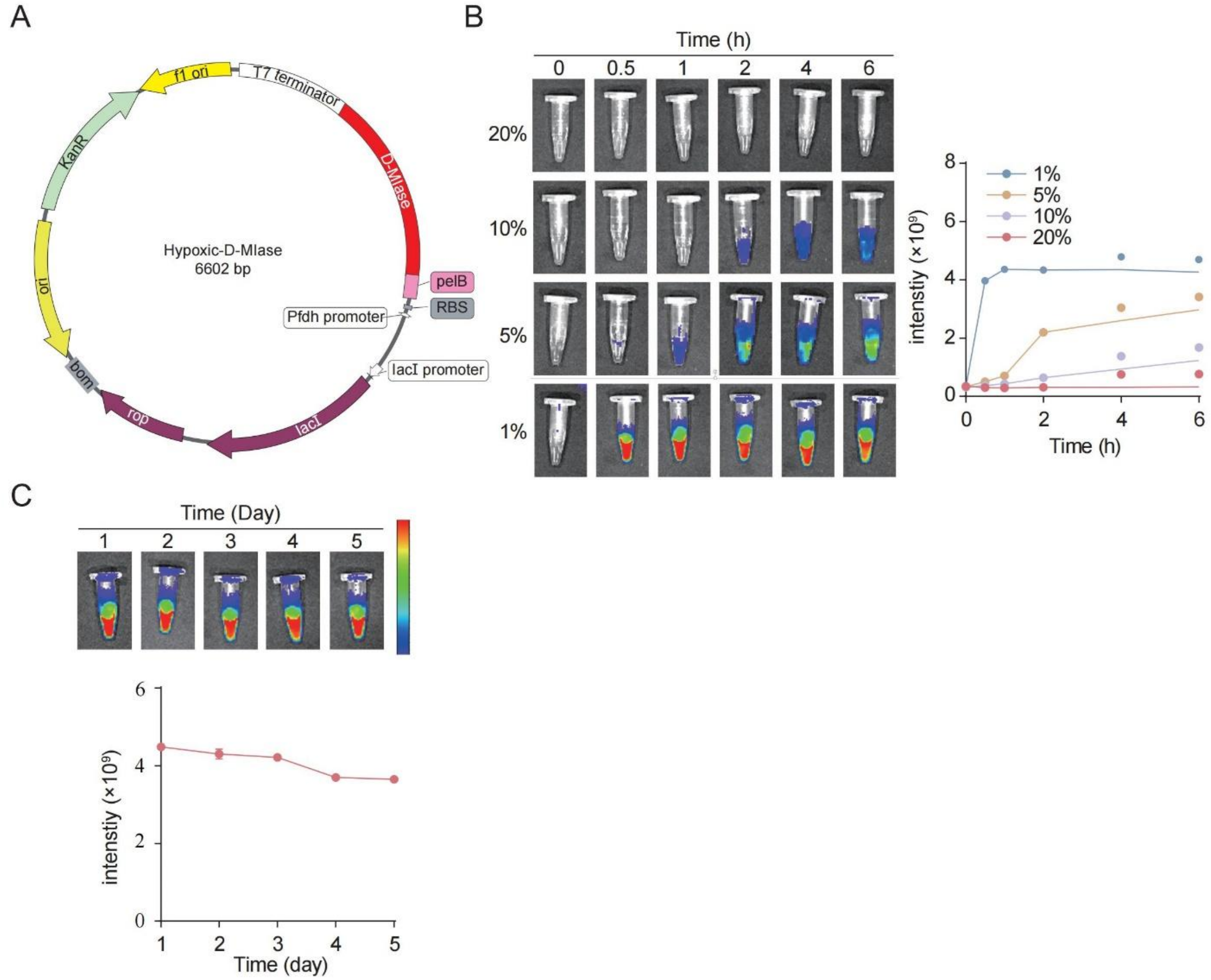

Figure S1. Visual observation of hypoxia-induced protein expression in engineered bacteria. (A) Scheme of the plasmid map for pET28a-hypoxic-D-MIase construction (The illustration was created using SnapGene® software (from Dotmatics; available at snapgene.com)). **(B)** Representative fluorescence images and quantitative analysis of mCherry in MG-mCherry at different time points after hypoxia induction. n = 5 samples/group. **(C)** Representative fluorescence images and quantitative analysis of mCherry in MG-mCherry at different time points after hypoxia induction. n = 5 samples/group. p values were calculated using two-way ANOVA following Fisher's LSD test for multiple comparisons. Data are mean ± SEM.

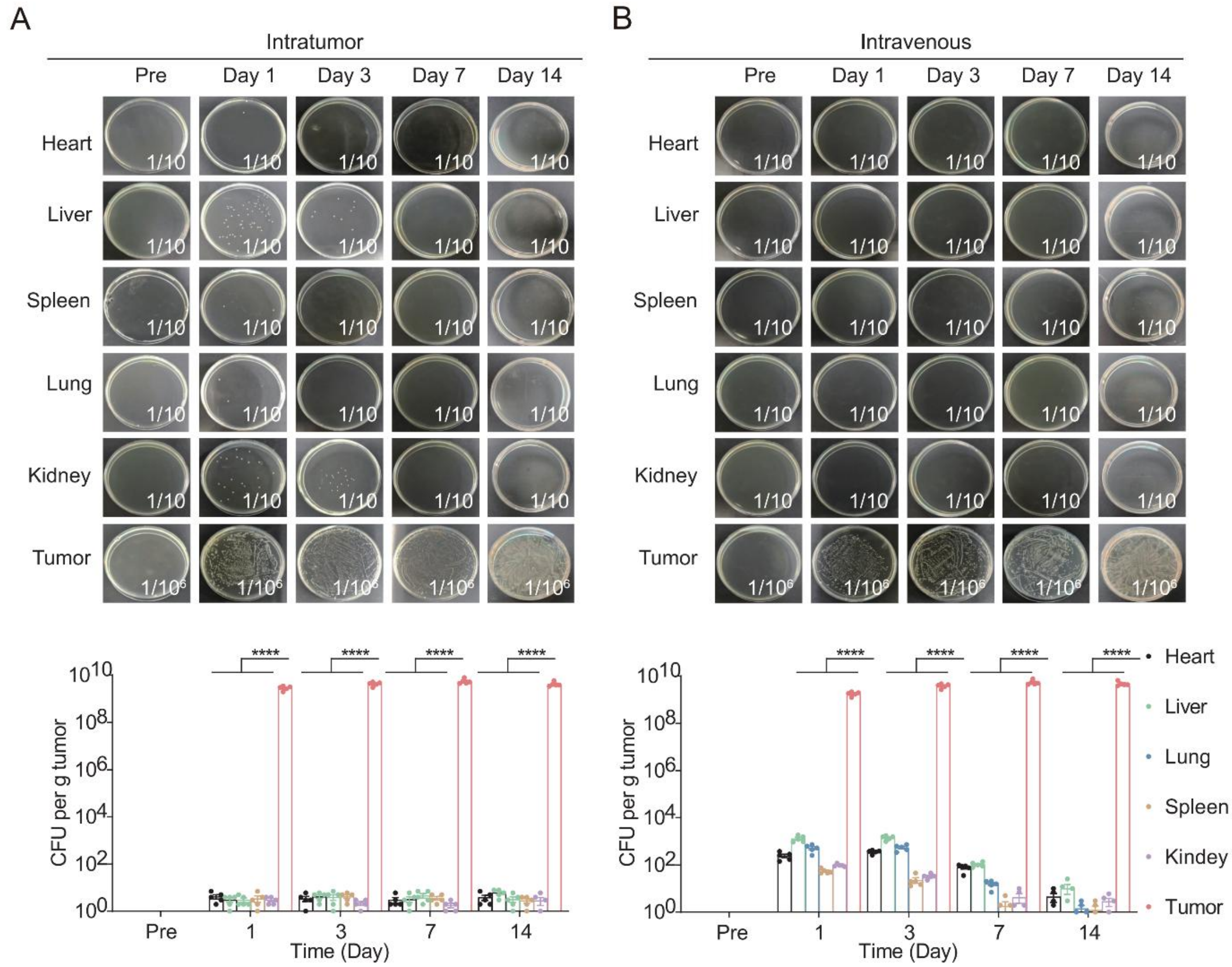

Figure S2. Tumor-specific colonization of engineered bacteria. (A) Representative photographs (up) and quantification of LB agar plates (down) spread with tissue homogenates of tumors and major organs after intratumoral injection of MG-MIase. **(B)** Representative photographs (up) and quantification of LB agar plates (down) spread with tissue homogenates of tumors and major organs after intravenous injection of MG-MIase. $n = 5$ samples/group. p values were calculated using one-way ANOVA with Tukey's multiple comparison test. Data are mean ± SEM. ****$p < 0.0001$.

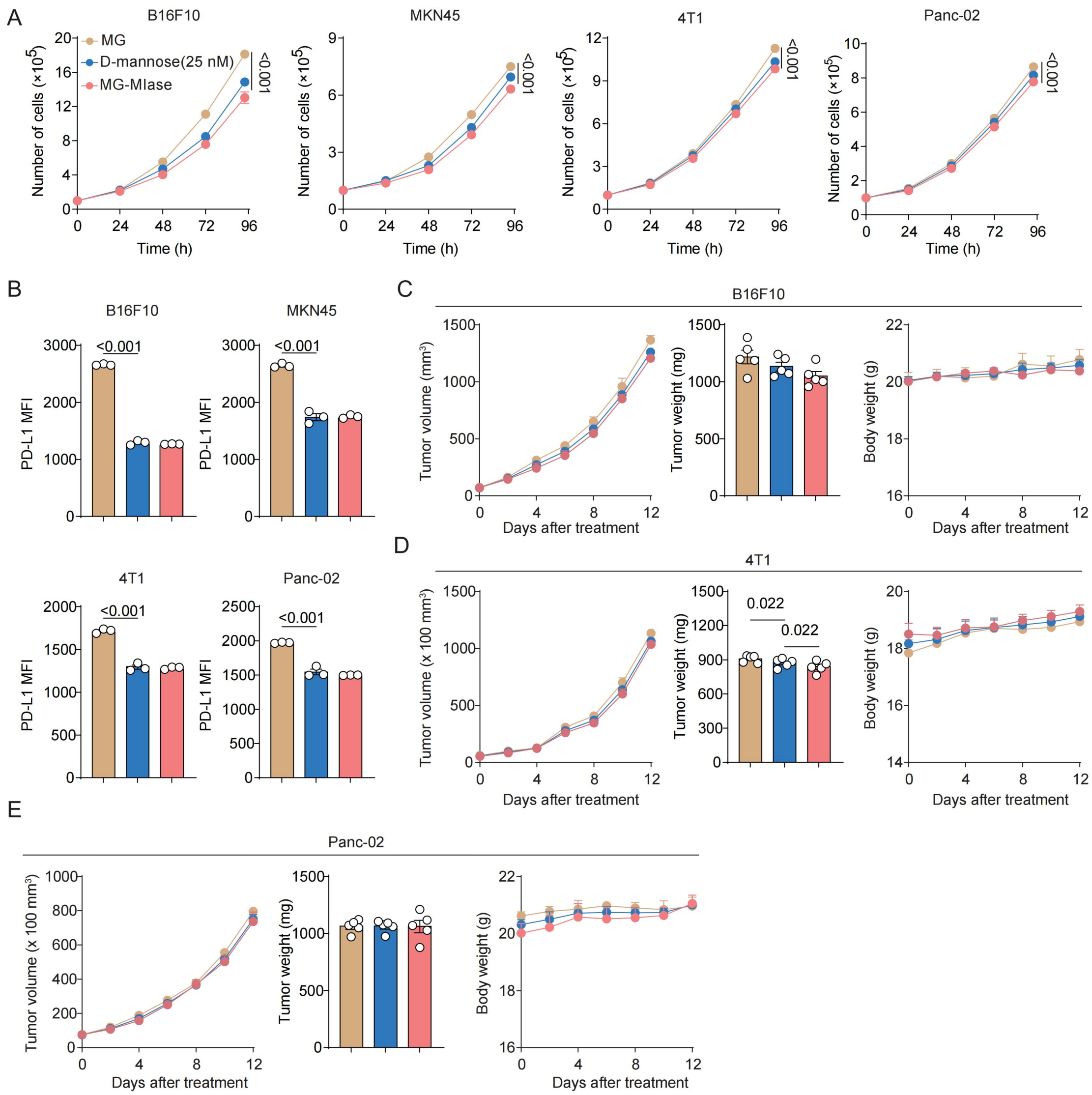

Figure S3.MG-MIase impairs the growth of cancer cells. (A) Growth curves of tumor cells after co-incubation with MG, D-mannose, or MG-MIase. n = 4 samples/group. **(B)** Quantitative analysis of the mean fluorescence intensity (MFI) of PD-L1 on tumor cells after incubation with MG, D-mannose, or MG-MIase. n = 3 samples/group. **(C-E)** Tumor growth curves, tumor weights, and changes in body weight of immunodeficient mice after treatment in B16F10 **(C)**, 4T1**(D)**, and Panc-02**(E)** tumor models. n = 5 samples/group. p values were calculated using unpaired two-sided Student's t test (**B-E**) and two-way ANOVA following Fisher's LSD test for multiple comparisons **(A, C-E)**. Data are mean ± SEM.

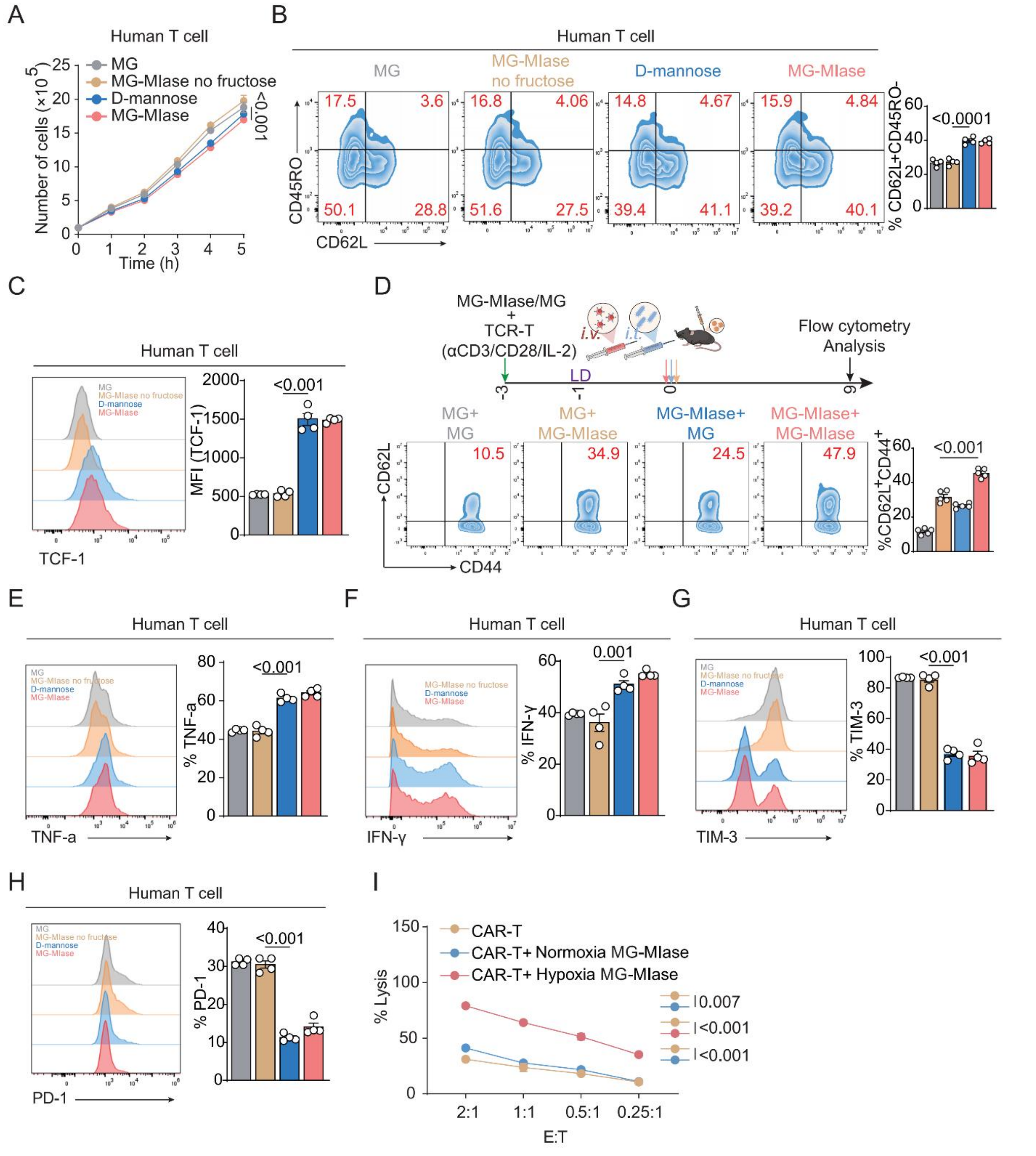

Figure S4. The effect of MG-MIase treatment on human T cells *in vitro*. (A) The numbers of human T cells expanded in MG, MG-MIase no fructose, D-mannose or MG-MIase. *n* = 4 samples/group. **(B)** Representative flow cytometry plots and quantitative analysis of CD62L⁺CD45RO- expression in human T cells after co-culture with MG, MG-MIase no fructose, D-mannose or MG-MIase. *n* = 4 samples/group. **(C)** Representative flow cytometry plots and

quantitative analysis of TCF1 expression in human T cells after co-culture with MG, MG-MIase no fructose, D-mannose or MG-MIase. *n* = 4 samples/group. **(D)** Schematic of the animal experiment (Up). Representative flow cytometry plots and quantitative analysis of $CD44^{+}CD62L^{+}$ expression in murine T cells *in vivo* (Day 9) after treatment with MG or MG-MIase (Down). **(E-F)** Representative flow cytometry plots and quantitative analysis of exhaustion marker expression in human T cells after co-culture with MG, MG-MIase no fructose, D-mannose or MG-MIase. *n* = 4 samples/group. **(G-H)** Representative flow cytometry plots and quantitative analysis of IFN-γ and TNF-α expression in human T cells after co-culture with MG, MG-MIase no fructose, D-mannose or MG-MIase. *n* = 4 samples/group. **(I)** Under different oxygen conditions, the cytotoxicity of CAR-T cells with or without MG-MIase was evaluated by co-culturing them with HGC-Claudin18.2 cells at various effector **(E)** to target **(T)** ratios. *p* values were calculated using unpaired two-sided Student's *t* test (**B-G**) and two-way ANOVA following Fisher's LSD test for multiple comparisons **(A, H)**. Data are mean ± SEM.

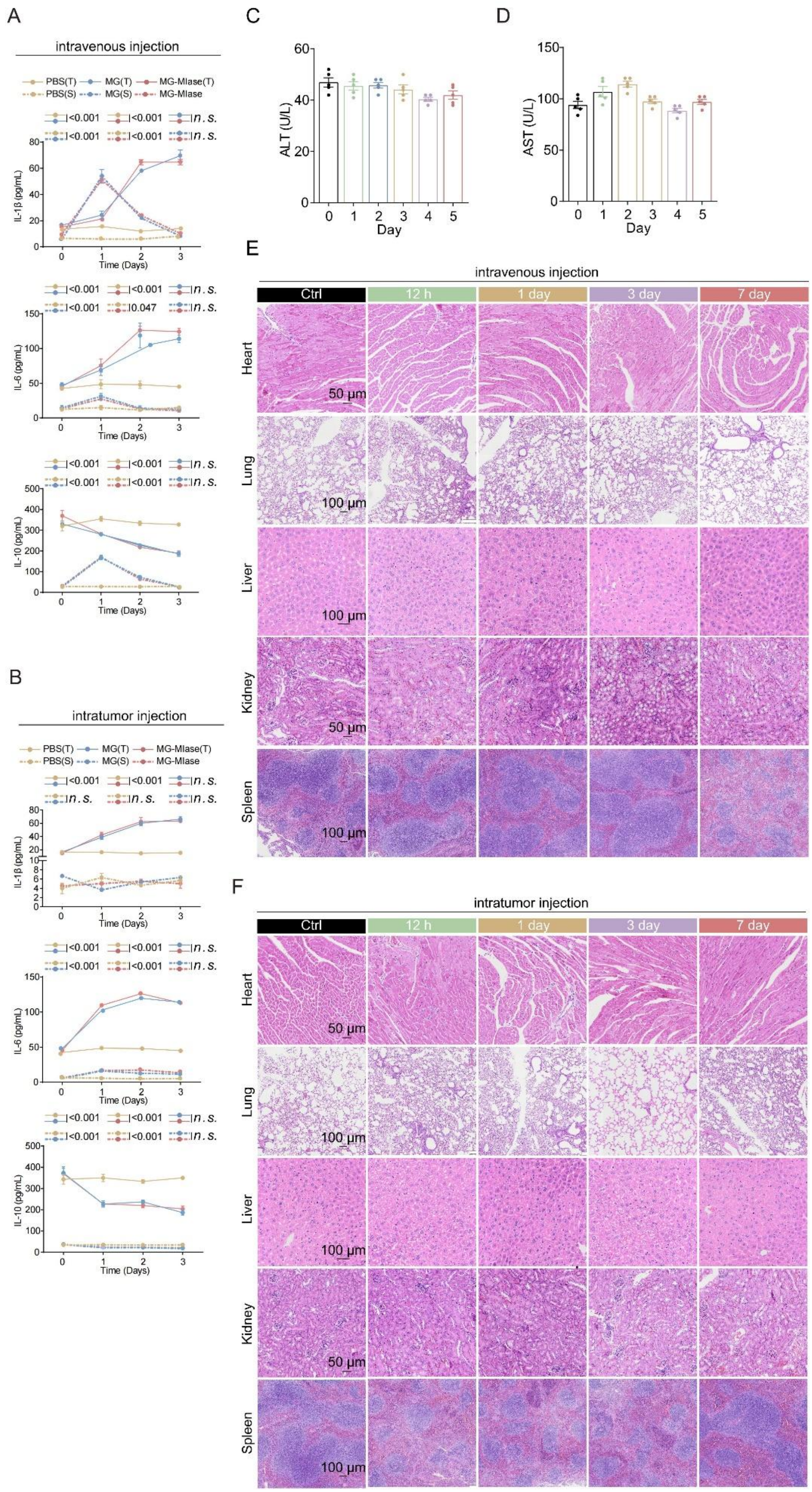
A
intravenous injection
PBS(T)
MG(T)
MG-MIase(T)
PBS(S)
MG(S)
MG-MIase
IL-1β (pg/mL)
IL-6 (pg/mL)
IL-10 (pg/mL)
Time (Days)
<0.001
0.047
n.s.
B
intratumor injection
C
ALT (U/L)
Day
D
AST (U/L)
E
intravenous injection
Ctrl
12 h
1 day
3 day
7 day
Heart
Lung
Liver
Kidney
Spleen
50 μm
100 μm
F
intratumor injection

Figure S5. In vivo safety evaluation of MG-MIase. (A-B) Line charts showing changes in serum and intratumoral levels of IL-1β, IL-6, and IL-10 in mice treated with MG-MIase via intravenous **(A)** or intratumoral **(B)** injection. "T" indicates tumor, and "S" indicates serum. AST **(C)** and ALT **(D)** levels in mice after intratumoral injection of MG-MIase. **(E-F)** Representative H&E staining images of various organs of mice treated with MG-MIase via intravenous **(E)** or intratumoral **(F)** injection at different time points (n = 5 mice). The scale bar was 100 μm and 50 μm. p values were calculated using two-way ANOVA following Fisher's LSD test for multiple comparisons. Data are mean ± SEM. *n.s.*, not significant.

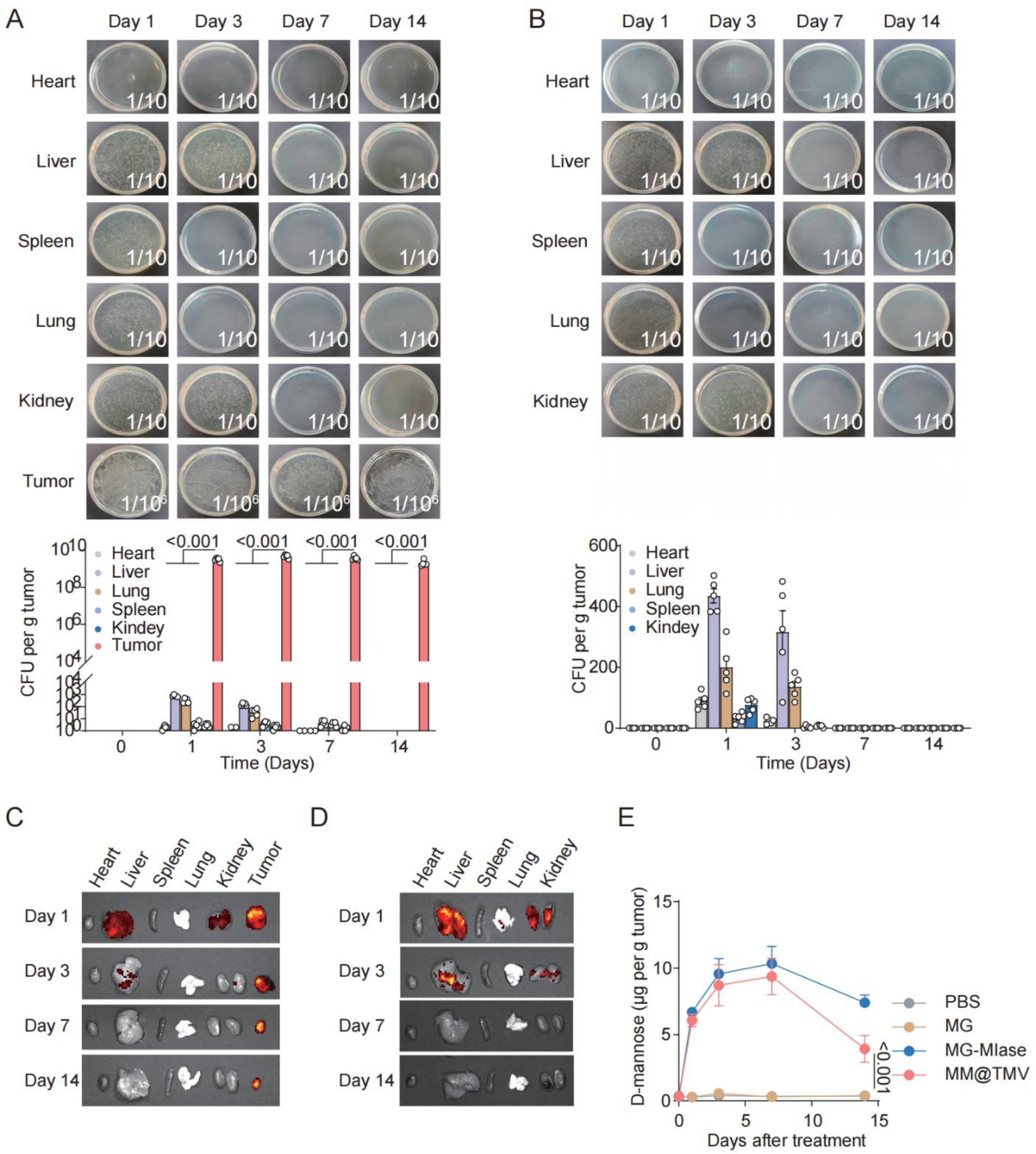

Figure S6. Tumor-specific colonization of MM@TMV. (A) Representative images and quantification of LB agar plates seeded with tissue homogenates from tumors and major organs

of tumor-bearing mice after intravenous injection of MM@TMV. **(B)** Representative images and quantification of LB agar plates seeded with tissue homogenates from major organs of healthy mice after intravenous injection of MM@TMV. **(C)** Representative fluorescence images showing the colonization of MM@TMV in various organs after intravenous injection. Tumor-bearing mice **(C)** and normal mice **(D)**. **(E)** After intravenous injection of MM@TMV, D-mannose levels in tumor tissue homogenates were quantified at different time points. (n = 5 mice/groups). p values were calculated using two-way ANOVA following Fisher's LSD test for multiple comparisons. Data are mean ± SEM.

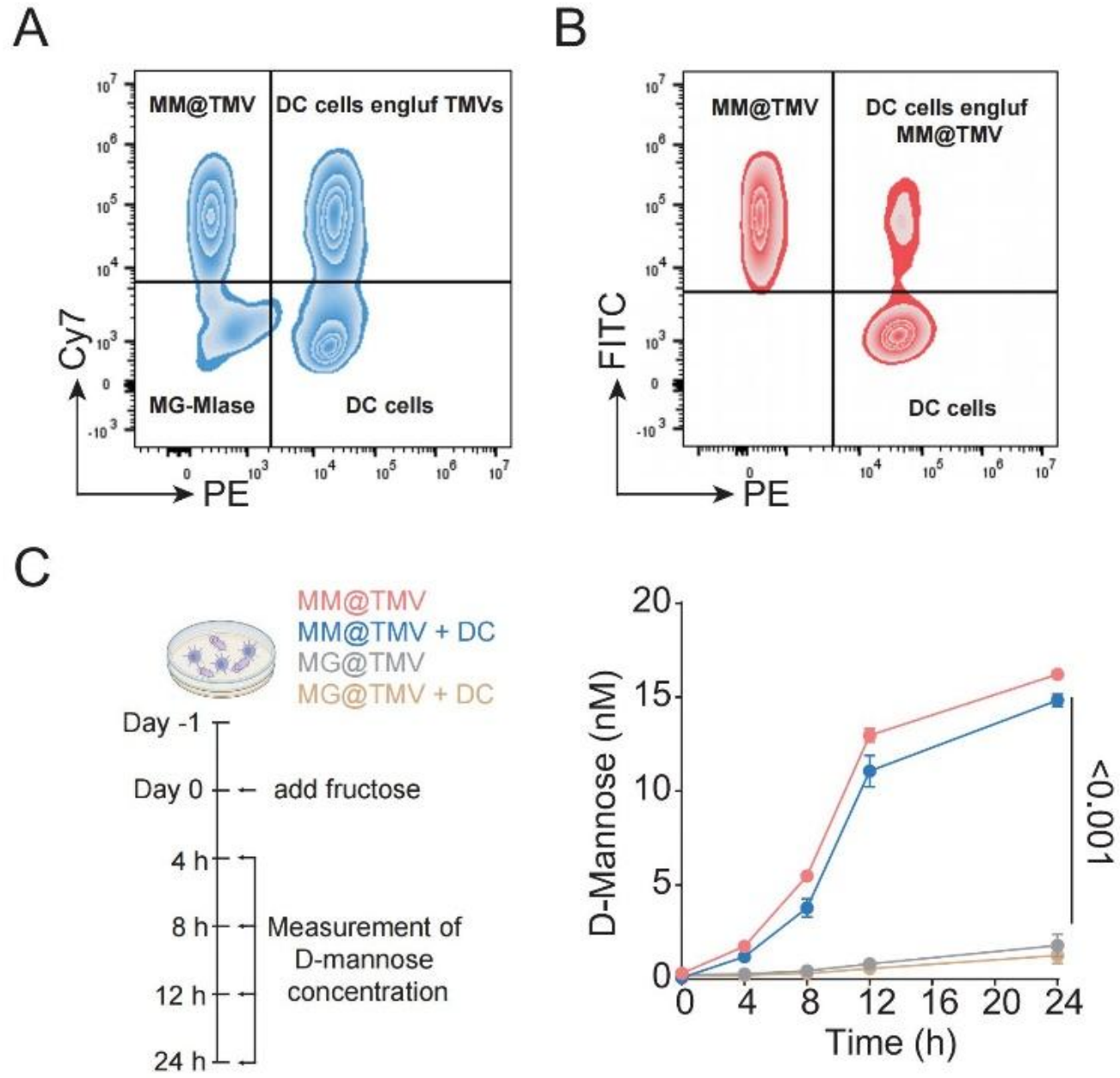

Figure S7. APCs selectively uptake TMVs while avoiding damage to MG-MIase. In MM@TMV, MG-MIase was labeled with FITC and the TMVs with the near-infrared fluorescent dye Cyanine7 (Cy7), followed by co-incubation with BMDCs. **(A)** DCs could efficiently uptake TMVs from MM@TMV. **(B)** DCs rarely engulf the bulky MM@TMV, thereby preserving the normal function of MG-MIase. **(C)** MM@TMV or MG@TMV were co-cultured with DCs or cultured alone, and after 1 day, 25 nM fructose was added to the medium, and the D-mannose concentration in the supernatant was measured every 2 hours. *p* values were calculated using two-way ANOVA following Fisher's LSD test for multiple comparisons. Data are mean ± SEM.

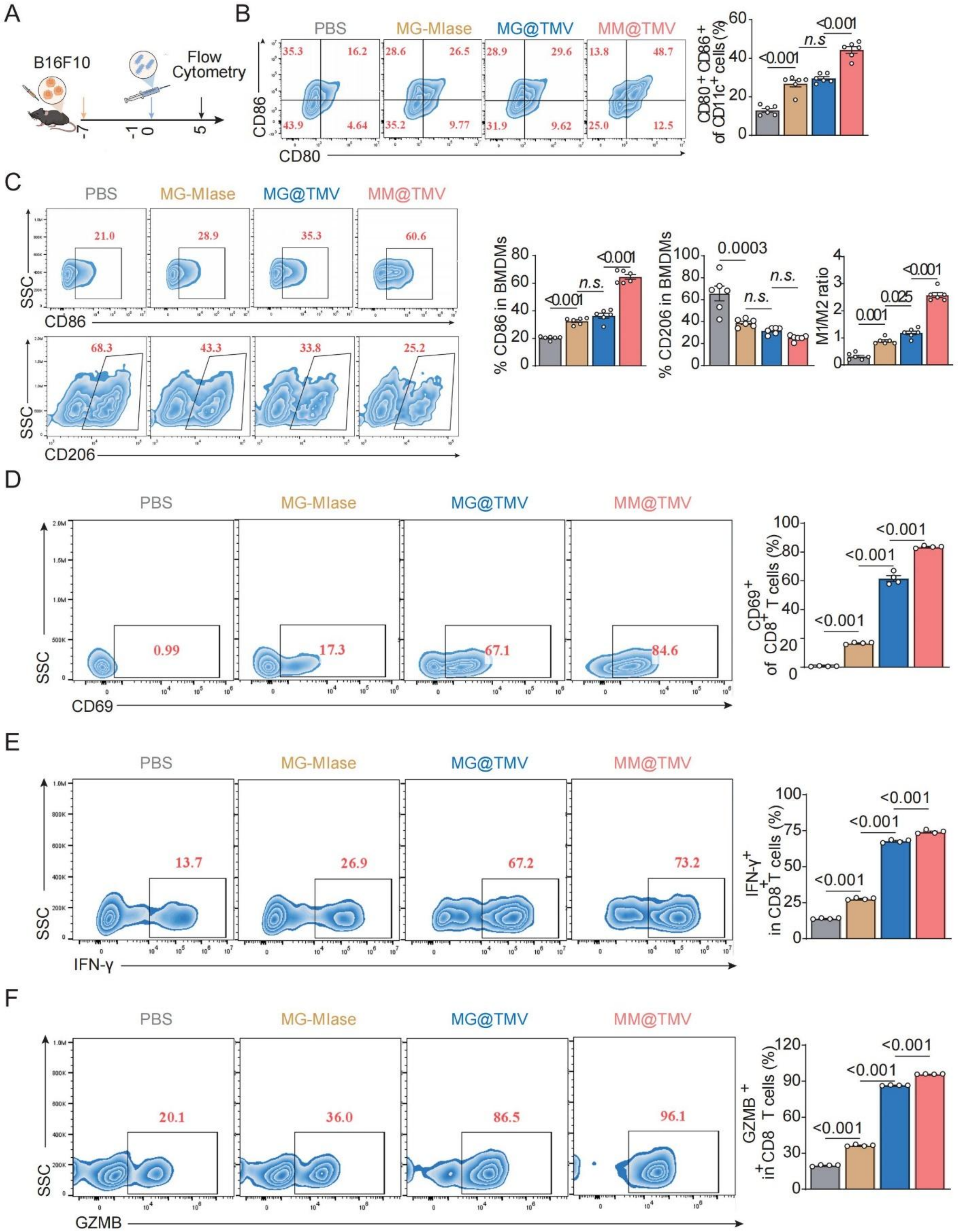

Figure S8.MM@TMV exhibited superior in vivo anti-tumor efficacy. (A) Experimental process. n = 6 samples/group. **(B)** Representative flow cytometry plots and quantitative analysis

of CD80$^+$CD86$^+$ expression in DCs in tumors. **(C-D)** Representative flow cytometry plots and quantitative analysis of CD86$^+$, CD206$^+$ expression in macrophages and the M1/M2 ratio in tumors. **(E-F)** Representative flow cytometry plots and quantitative analysis of CD69$^+$ T cells **(E)** and IFN-γ^+ T cells **(E)** and Granzyme B$^+$ T cells. **(F)** in tumors. *p* values were calculated using unpaired two-sided Student's t test (**B-E**) and two-way ANOVA following Fisher's LSD test for multiple comparisions **(A, C-E)**. *p* values were calculated using one-way ANOVA with Tukey's multiple comparison test **(B-F)**. Data are mean ± SEM. *n.s.*, not significant.

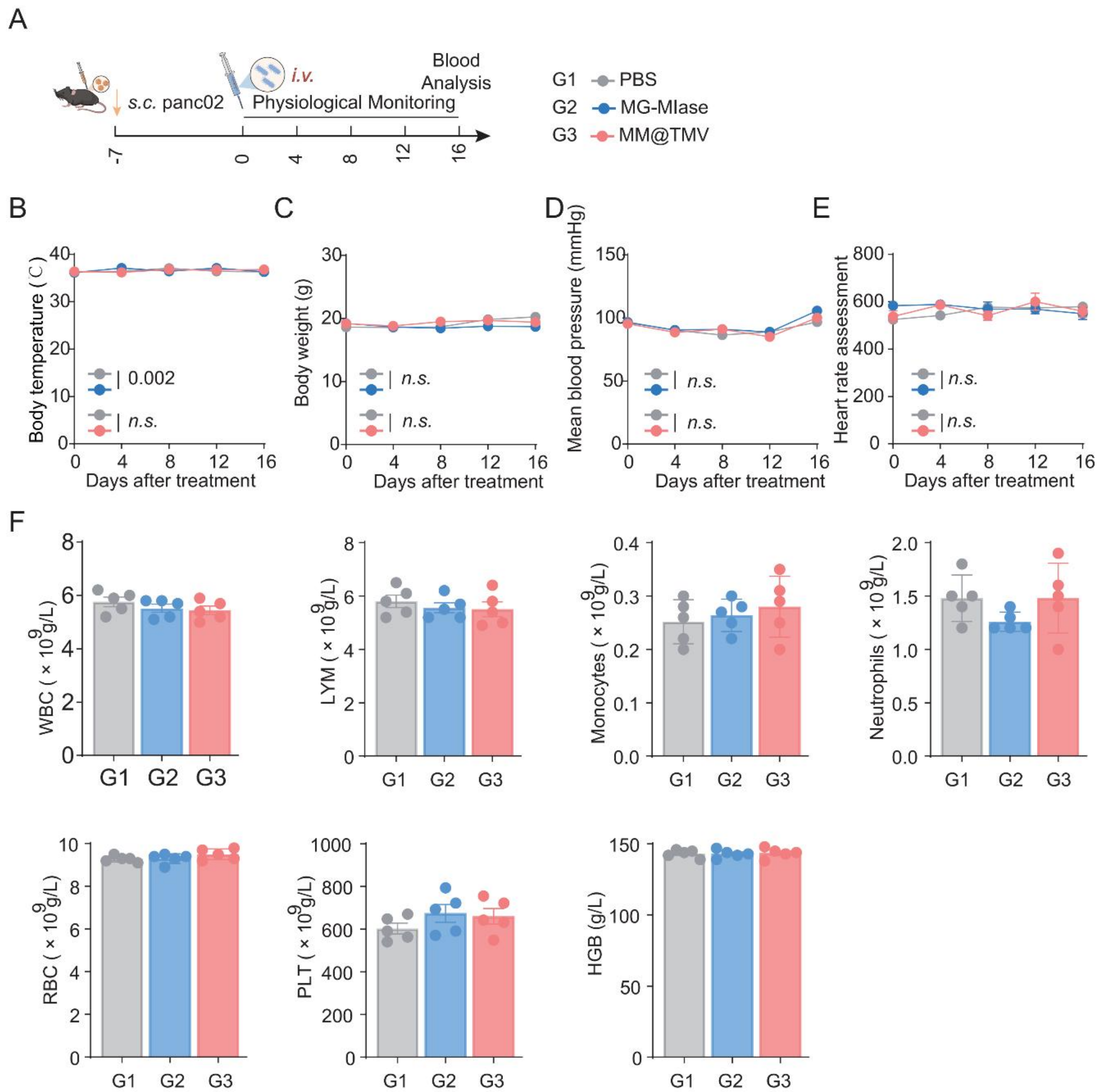

Figure S9. Comprehensive safety profile evaluation of MM@TMV. (A) Schematic illustration of the safety evaluation study in Panc-02 tumor-bearing mice. Mice received intravenous (i.v.) injections at the indicated time points, followed by physiological monitoring and blood analysis. **(B)** Body temperature. **(C)** Body weight changes. **(D)** Mean blood pressure. **(E)** Heart rate. (F) Blood cell counts on Day 16, including white blood cells (WBC), lymphocytes (LYM), monocytes (MON), neutrophils (NEU), red blood cells (RBC), platelets (PLT), and hemoglobin (HGB), to evaluate hematological toxicity. p values were calculated using unpaired two-sided Student's t test. Data are mean ± SEM. *n.s.*, not significant.

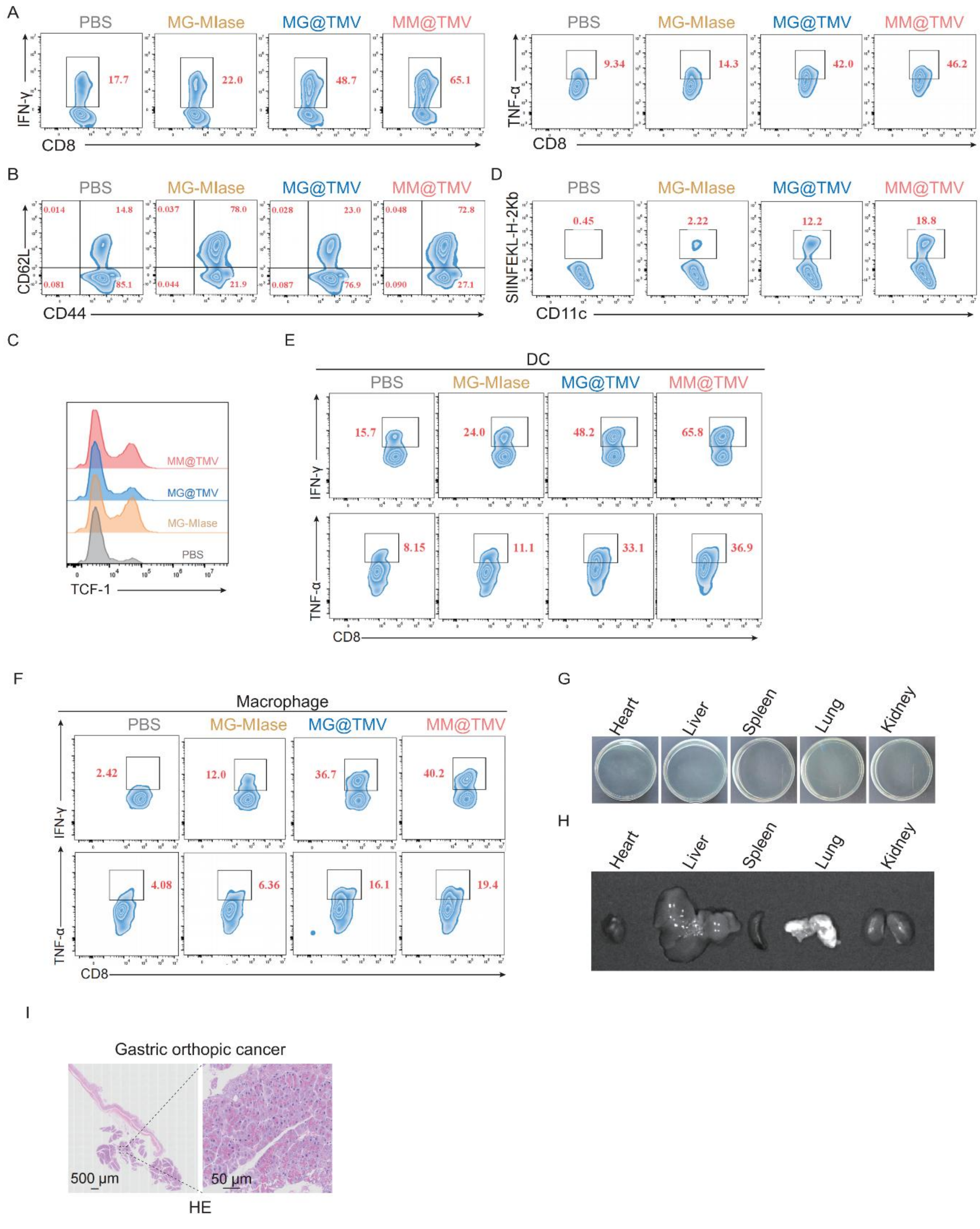

Figure S10. MM@TMV significantly enhanced the antitumor efficacy of TCR-T cells. Related to Fig 5. (A) Representative flow cytometry plots of intracellular IFN-γ and TNF-α in $CD8^+$ T cells. **(B)** Representative flow cytometry plots of the percentage of $CD44^+CD62L^+$ cells

in $CD8^+$ T cells. **(C)** Representative histograms analysis the expression of MFI in $CD8^+$ T cells. **(D)** Representative flow cytometry plots of the percentage of SIINFEKL-H-2Kb cells in $CD8^+$ T cells. **(E, F)** Representative flow cytometry plots of intracellular IFN-γ and TNF-α in $CD8^+$ T cells after co-incubation with BMDCs **(E)** or BMDMs **(F)**. **(G)** Representative images of colonies on LB agar plates seeded with tissue homogenates from major organs of cured mice. **(H)** Representative fluorescence images showing bacterial colonization in various organs of cured mice.

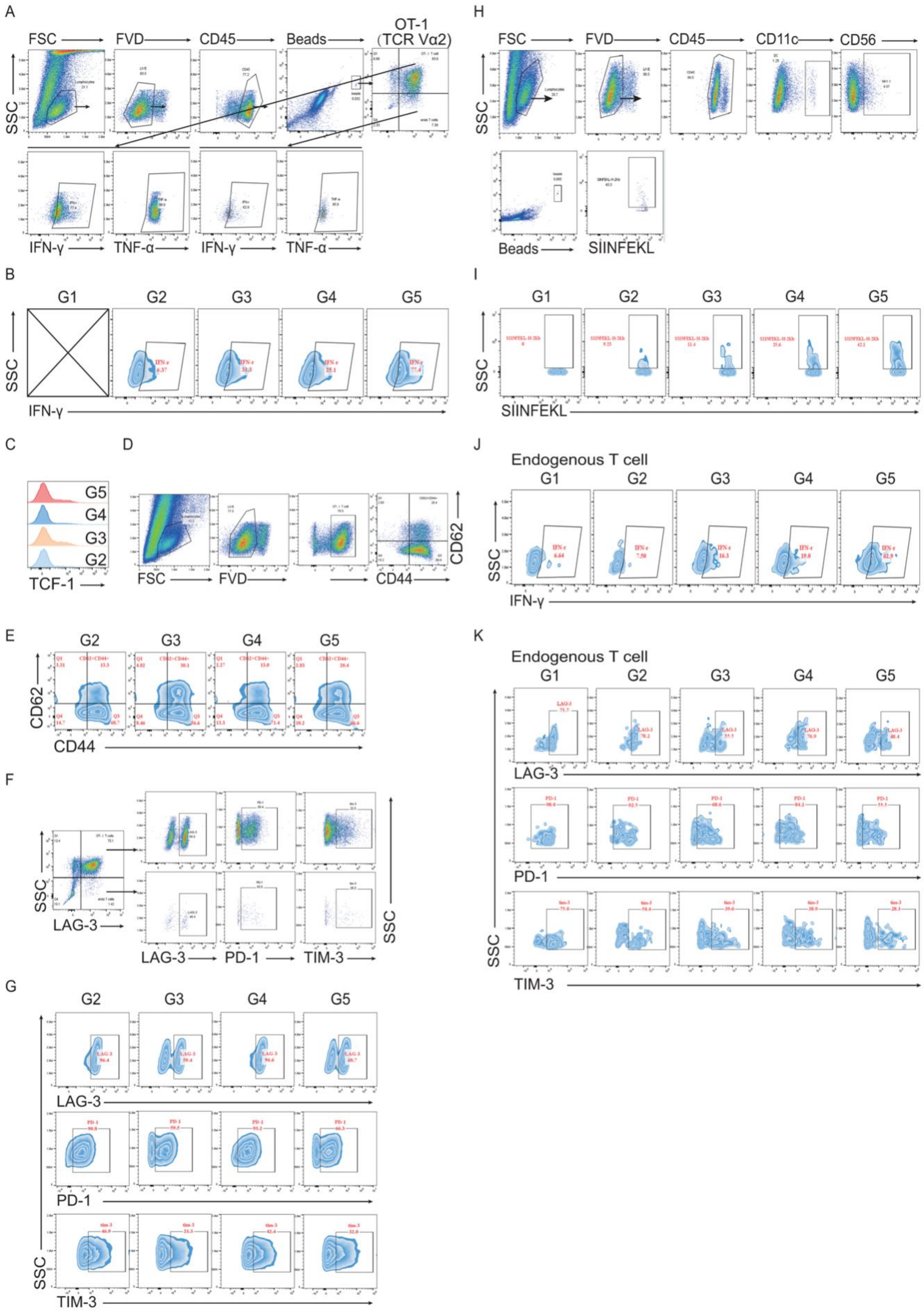
A
FSC
FVD
CD45
Beads
OT-1 (TCR Vα2)
SSC
IFN-γ
TNF-α
IFN-γ
TNF-α
B
G1
G2
G3
G4
G5
SSC
IFN-γ
C
G5
G4
G3
G2
TCF-1
D
FSC
FVD
CD44
CD62
E
G2
G3
G4
G5
CD62
CD44
F
SSC
LAG-3
LAG-3
PD-1
TIM-3
SSC
G
G2
G3
G4
G5
LAG-3
PD-1
SSC
TIM-3
H
FSC
FVD
CD45
CD11c
CD56
SSC
Beads
SIINFEKL
I
G1
G2
G3
G4
G5
SSC
SIINFEKL
J
Endogenous T cell
G1
G2
G3
G4
G5
SSC
IFN-γ
K
Endogenous T cell
G1
G2
G3
G4
G5
LAG-3
PD-1
SSC
TIM-3

Figure S11. Mechanistic study of the significant enhancement of TCR-T cell antitumor efficacy by MM@TMV in vivo. Related to Fig 6. (A) FCM gating strategy for the analysis of tumor infiltrating TCR-T cells in tumors. **(B)** Representative flow cytometry plots of intracellular IFN-γ in TCR-T cells. **(C)** Representative histograms analysis of TCF-1 expression in TCR-T cells. **(D)** FCM gating strategy for the $CD62L^{+}CD44^{+}$ cells in TCR-T cells in tumors. **(E)** Representative flow cytometry plots of the percentage of $CD44^{+}$CD62L+ in TCR-T cells. **(F)** FCM gating strategy for the analysis of exhaustion markers in tumor-infiltrating TCR-T cells in tumors. **(G)** Representative flow cytometry plots of the expression of exhaustion markers TIM-3, PD-1, and LAG-3 in TCR-T cells in tumors. **(H)** FCM gating strategy for the analysis of endogenous T cells, DCs, and NK cells in tumors. **(I)** Representative flow cytometry plots of SIINFEKL-H-2Kb presentation in DCs in tumors. **(J)** Representative flow cytometry plots of intracellular IFN-γ in endogenous T cells. **(K)** Representative flow cytometry plots of the expression of exhaustion markers TIM-3, PD-1, and LAG-3 in endogenous T cells in tumors.

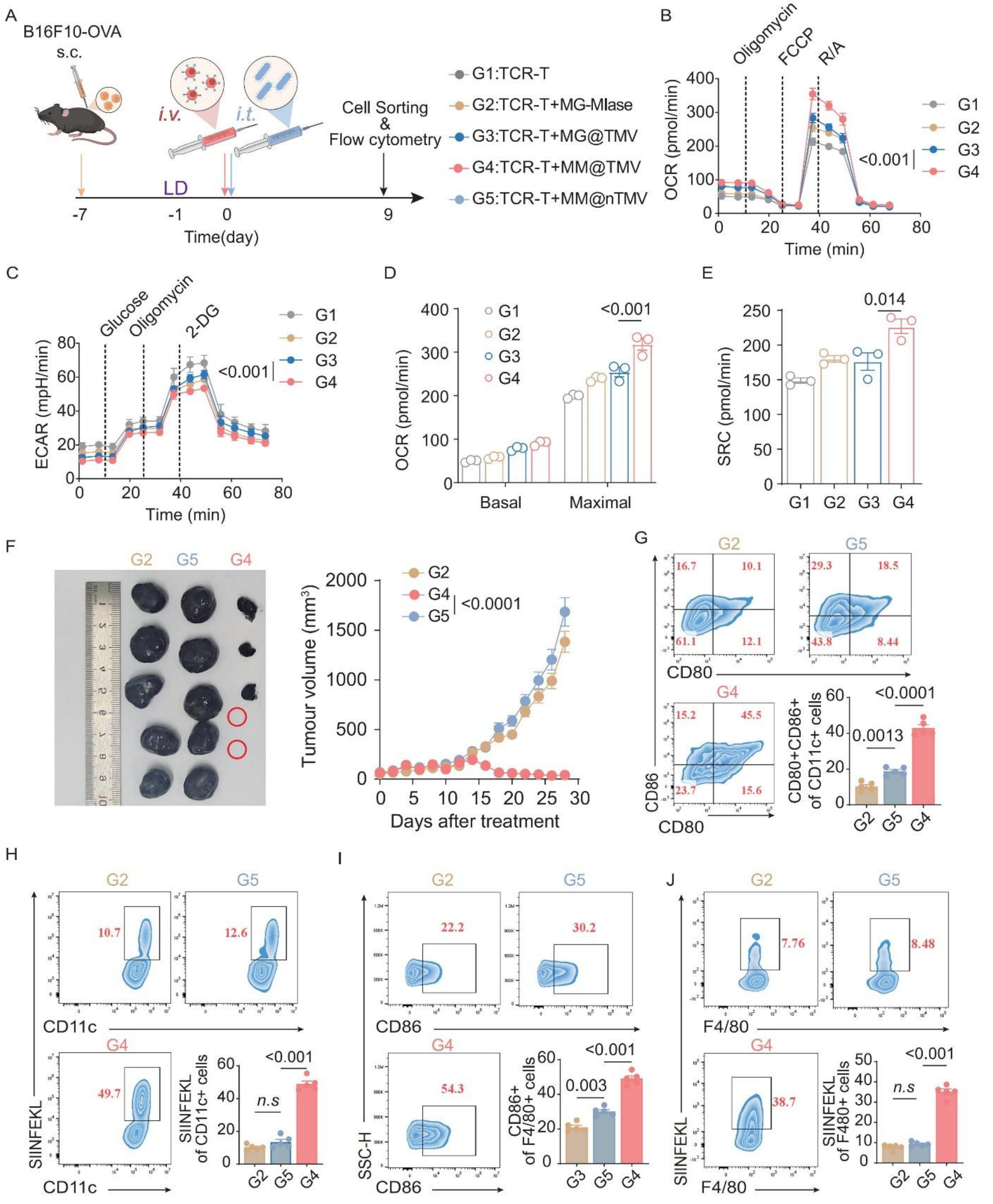

Figure S12. Related to Fig 6. (A) Schematic of the experimental workflow. B16F10-OVA tumors were implanted subcutaneously (s.c.), followed by adoptive transfer of TCR-T cells and intratumoral or intravenous administration of the indicated bacterial formulations. Groups: G1,

TCR-T; G2, TCR-T+MG-MIase; G3, TCR-T+MG@TMV; G4, TCR-T+MM@TMV; G5, TCR-T+MM@nTMV (TMVs lacking OVA and derived solely from B16F10 tumor cell membranes). **(B–E)** Metabolic analysis of tumor-infiltrating TCR-T cells by Seahorse mitochondrial stress test. **(B)** Oxygen consumption rate (OCR). **(C)** Extracellular acidification rate (ECAR). **(D)** Basal and maximal OCR. **(E)** Spare respiratory capacity (SRC). **(F)** Representative tumor images (left) and tumor growth curves (right). **(G)** Representative flow cytometry plots and quantitative analysis of $CD80^+ CD86^+$ expression in DCs in tumors. **(H)** Representative flow cytometry plots and quantitative analysis of SIINFEKL expression in DCs in tumors. **(I)** Representative flow cytometry plots and quantitative analysis of CD86 expression in macrophages in tumors. **(J)** Representative flow cytometry plots and quantitative analysis of SIINFEKL expression in macrophages in tumors. *p* values were calculated using unpaired two-sided Student's *t* test (**B-D, F**) and two-way ANOVA following Fisher's LSD test for multiple comparisons **(E, G-J)**. Data are mean ± SEM. *n.s.*, not significant.

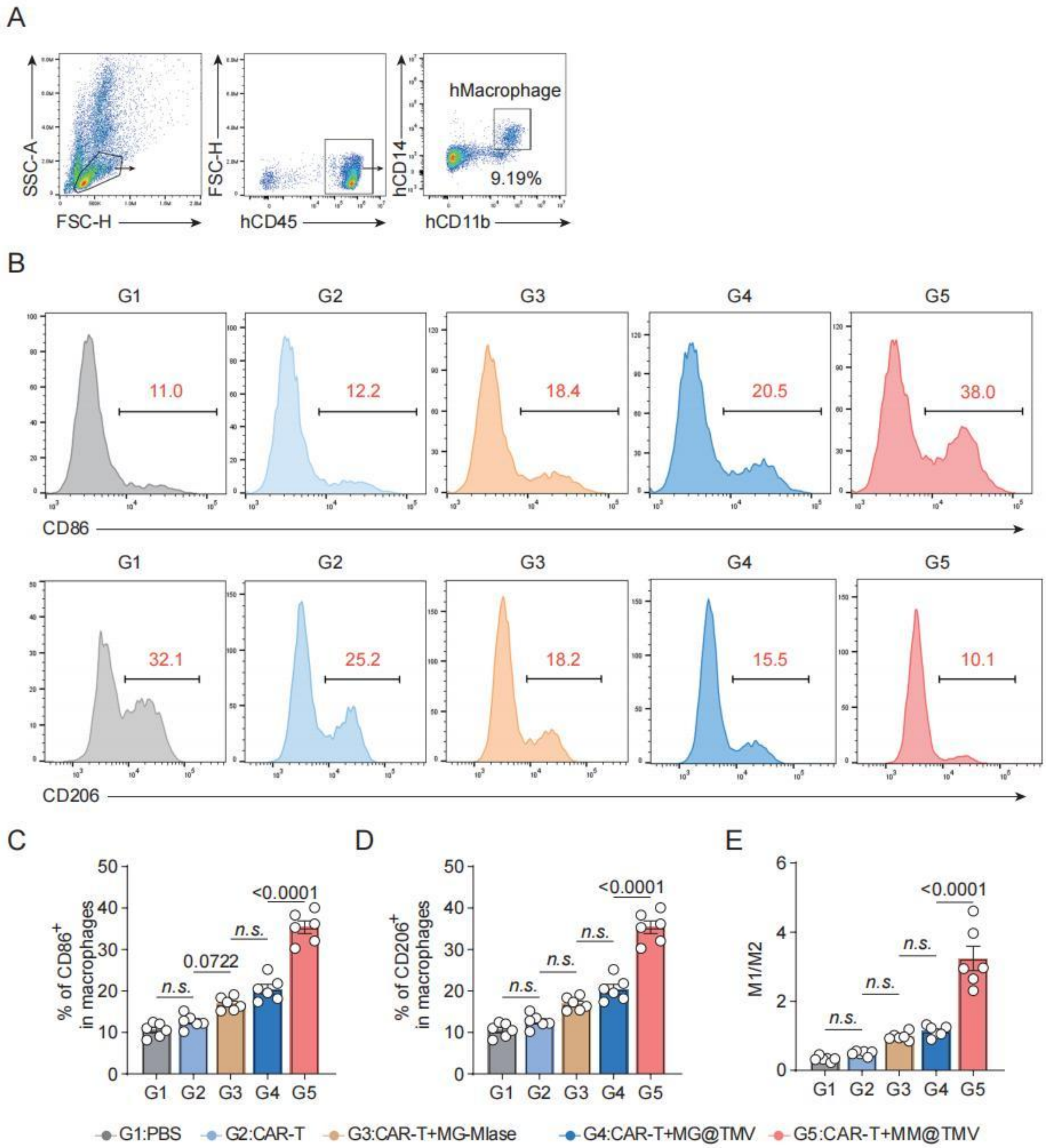

Figure S13. Related to Fig 7. (**A**) Representative flow cytometry gating of peripheral human macrophages before treatment allocation. (**B**) Percentages of human $CD45^+$ cells, $CD8^+$ T cells and $CD11b^+$cells before dosing in the peripheral blood of mice (referenced in **Figure 7B**) prior to dosing; (**C**) Human M1 like and M2 like macrophages were identified by flow cytometry as $hCD45^+CD11b^+CD14^+CD86^+/CD206^+$ cells, respectively. (**D**) Quantitative analysis of $CD86^+$ and $CD206^+$ macrophage population and M1/M2 ratio in BMDMs. n = 6 samples/group. p values

were calculated using unpaired two-sided Student's *t* test. Data are mean ± SEM. *n.s.*, not significant.

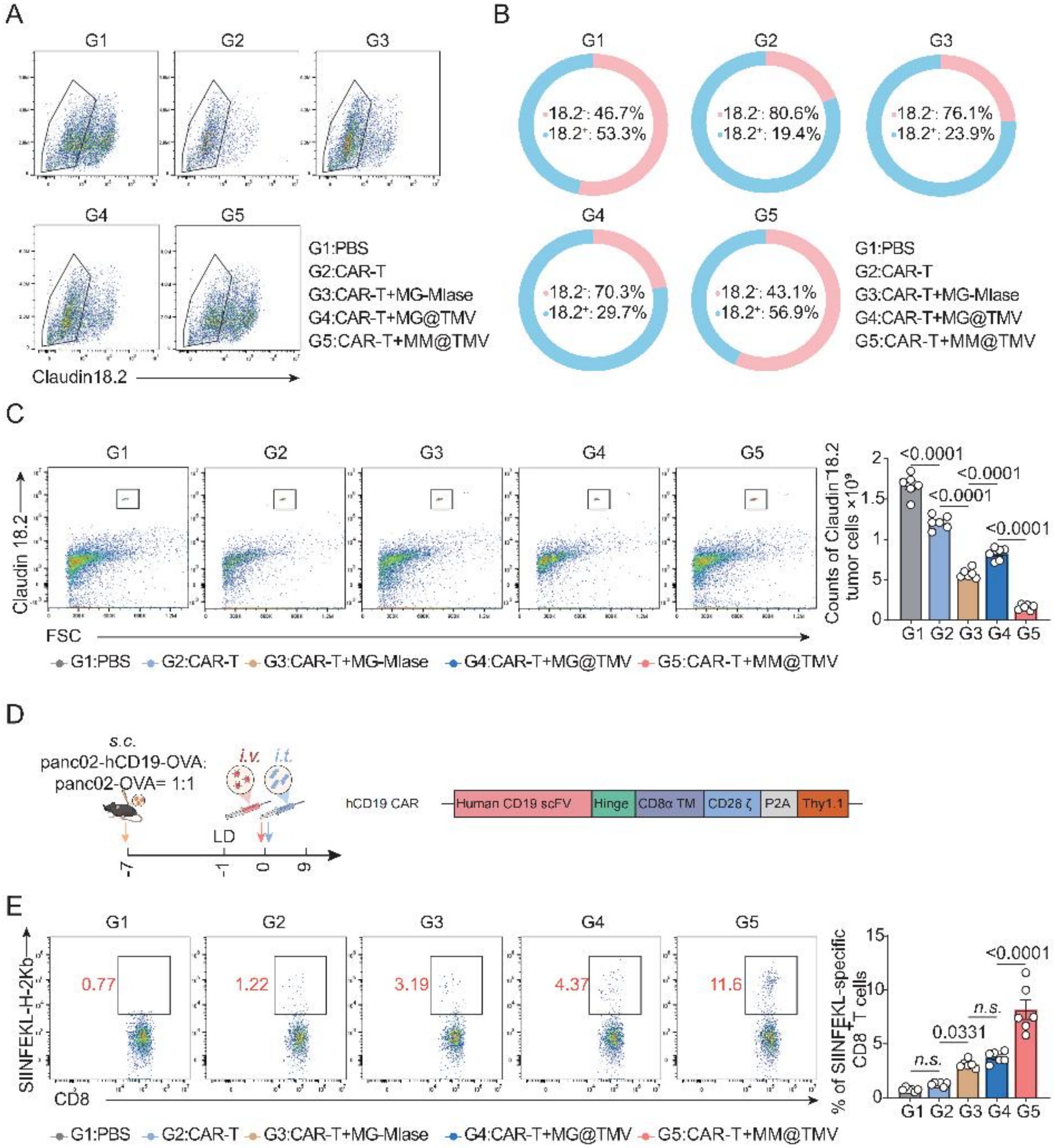

Figure S14. MM@TMV restricts antigen negative outgrowth and promotes antigen spreading during CAR-T therapy. **(A)** Representative flow cytometry plot showing baseline Claudin18.2 expression in tumor cells before implantation; **(B)** Representative flow cytometry plots and quantification of Claudin18.2 negative tumor cells after the indicated treatments; **(C)** Experimental scheme of the mixed Panc02-hCD19-OVA/Panc02-OVA tumor model and the structure of the hCD19 CAR construct used to assess antigen spreading; **(D)** Representative flow

cytometry plots and quantification of SIINFEKL specific CD8+ T cells in tumors after treatment. p values were calculated using unpaired two-sided Student's t test. Data are mean ± SEM. *n.s.*, not significant.

Table S1 D-MIase component sequences

Element name	sequence
pelB signal	GHRRLGSEEQTSSSGRQQVFH
D-mannose isomerase	MTLWTARAAHRAWLDAEARRLVDFAAAADHPEHGFAWLDGSGAP LPEQGVHTWITCRVTHVAALAHLEGIPGASALADHGLRALAGPLRD PEHDGWFTALDSRGTVADSRKEAYQHAFVLLAAASATVAGRPGAR ELLDAAAAVIEQRFWEEETGRCRESWDAAWHADEPYRGANSNMHL VEAFLAAFDATGDRVWAERALRIAHFFVHEVAAPRDWRLPEHFTPD WQVVADYNTDDRAHPFRPYGVTVGHVLEWARLLVHVEAALPDPPS WLLADAEAMFAAAVARGWSVDGTEGFVYTLDYDDTPVVRSRMH WVVAEAISAAAVLGQRTGDERYEHWYRTWWDHAATYFVDTVQGS WHHELDPTLAPPPGGTWSGKPDVYHAYQATRLPLLPLAPSLAGALA TVG

Table S2 anti-Claudin18.2-CAR component sequences

Element name	sequence
CD8 signal sequence	MALPVTALLLPLALLLHAARP
Anti-Claudin18.2-scfv	QVQLQESGPGLVKPSQTLSLTCTVSGGSISSGYNWHWIRQPPGKGLE WIGYIHYTGSTNGGGGSGGGGSGGGGYNPALRSRVTISVDTSKNQF SLKLSSVTAADTAVYYCARIYNGNSFPYWGQGTTVTV
Human CD8 Hinge + Transmembrane	ALSNSIMYFSHFVPVFLPAKPTTTPAPRPPTPAPTIASQPLSLRPEASRP AAGGAVHTRGLDLQGLLGLLLGLVLLVLLL
Human CD28z	MLVAYDNAVNLSCKYSYNLFSREFRASLHKGLDSAVEVCVVYGNY SQQLQVYSKTGFNCDGKLGNESVTFYLQNLYVNQTDIYFCKIEVMY PPPYLDNEKSNGTIIHVKGMKWKALFTAAILQAQLPITEAQSFGLLDP KLCYLLDGILFIYGVILTALFLRVKFSRSADAPAYQQGQNQLYNELN LGRREEYDVLDKRRGRDPEMGGKPQRRKNPQEGLYNELQKDKMAE AYSEIGMKGERRRGKGHDGLYQGLSTATKDTYDALHMQALPPR
P2A	RKRRSGSGATNFSLLKQAGDVEENPGP
Thy1.1	MNPAISVALLLSVLQVSRGQKVTSLTACLVNQNLRLDCRHENNTKD

NSIQHEFSLTREKRKHVLSGTLGIPEHTYRSRVTLSNQPYIKVLTLAN
FTTKDEGDYFCELQVSGANPMSSNKSISVYRDKLVKCGGISLLVQNT
